# Comparing Arabidopsis receptor kinase and receptor protein‐mediated immune signaling reveals BIK1‐dependent differences

**DOI:** 10.1111/nph.15497

**Published:** 2018-10-25

**Authors:** Wei‐Lin Wan, Lisha Zhang, Rory Pruitt, Maricris Zaidem, Rik Brugman, Xiyu Ma, Elzbieta Krol, Artemis Perraki, Joachim Kilian, Guido Grossmann, Mark Stahl, Libo Shan, Cyril Zipfel, Jan A. L. van Kan, Rainer Hedrich, Detlef Weigel, Andrea A. Gust, Thorsten Nürnberger

**Affiliations:** ^1^ Department of Plant Biochemistry Centre for Plant Molecular Biology Eberhard Karls University Tübingen Auf der Morgenstelle 32 D‐72076 Tübingen Germany; ^2^ Department of Molecular Biology Max‐Planck‐Institute for Developmental Biology Max‐Planck‐Str. 5 D‐72076 Tübingen Germany; ^3^ Center for Genomics & Systems Biology New York University 12 Waverly Place New York NY 10003 USA; ^4^ Centre for Organismal Studies & Excellence Cluster Cell Networks Heidelberg University Im Neuenheimer Feld 230 69120 Heidelberg Germany; ^5^ Institute for Plant Genomics & Biotechnology Texas A&M University College Station TX 77843 USA; ^6^ Plant Physiology and Biophysics Julius Maximilians University Würzburg Julius‐von‐Sachs‐Platz 2 97082 Würzburg Germany; ^7^ Department of Biophysics Institute of Biology Maria Curie‐Skłodowska University Akademicka 19 20‐033 Lublin Poland; ^8^ The Sainsbury Laboratory Norwich Research Park Norwich NR4 7UH UK; ^9^ Department of Plant Sciences University of Cambridge Cambridge CB2 3EA UK; ^10^ Analytics Unit Centre for Plant Molecular Biology Eberhard Karls University Tübingen Auf der Morgenstelle 32 D‐72076 Tübingen Germany; ^11^ Laboratory of Phytopathology Wageningen University 6708 PB Wageningen the Netherlands; ^12^ Department of Biochemistry University of Johannesburg Auckland Park South Africa

**Keywords:** Arabidopsis, immune receptor, immune signaling comparison, plant immunity, receptor kinase, receptor protein

## Abstract

- Pattern recognition receptors (PRRs) sense microbial patterns and activate innate immunity against attempted microbial invasions. The leucine‐rich repeat receptor kinases (LRR‐RK) FLS2 and EFR, and the LRR receptor protein (LRR‐RP) receptors RLP23 and RLP42, respectively, represent prototypical members of these two prominent and closely related PRR families.
- We conducted a survey of *Arabidopsis thaliana* immune signaling mediated by these receptors to address the question of commonalities and differences between LRR‐RK and LRR‐RP signaling.
- Quantitative differences in timing and amplitude were observed for several early immune responses, with RP‐mediated responses typically being slower and more prolonged than those mediated by RKs. Activation of RLP23, but not FLS2, induced the production of camalexin. Transcriptomic analysis revealed that RLP23‐regulated genes represent only a fraction of those genes differentially expressed upon FLS2 activation. Several positive and negative regulators of FLS2‐signaling play similar roles in RLP23 signaling. Intriguingly, the cytoplasmic receptor kinase BIK1, a positive regulator of RK signaling, acts as a negative regulator of RP‐type immune receptors in a manner dependent on BIK1 kinase activity.
- Our study unveiled unexpected differences in two closely related receptor systems and reports a new negative role of BIK1 in plant immunity.

## Introduction

Metazoan and plant pattern recognition receptors (PRRs) are instrumental to pathogen sensing and activation of innate immune defenses in response to attempted microbial infections (Böhm *et al*., 2014a; Macho & Zipfel, 2014). Such receptors are germline‐encoded and detect molecules (‘patterns’) that are characteristic of whole classes of microbes (Boutrot & Zipfel, 2017). Detection of these molecules, called pathogen‐ or microbe‐associated molecular patterns (PAMPs/MAMPs), leads to a set of responses collectively referred to as pattern‐triggered immunity (PTI) (Monaghan & Zipfel, 2012; Böhm *et al*., 2014a; Saijo *et al*., 2018). Plant PRRs can be subdivided into those that recognize carbohydrate patterns and those that sense proteinaceous patterns (Gust *et al*., 2012; Monaghan & Zipfel, 2012; Böhm *et al*., 2014a; Boutrot & Zipfel, 2017). The recognition of fungal chitin or of bacterial peptidoglycan and lipopolysaccharide is mediated by either lysin‐motif‐type receptor complexes or lectin S‐DOMAIN receptor kinases. Plant leucine‐rich repeat (LRR) receptor proteins (RPs) and LRR‐receptor kinases (RKs) have been shown to sense multiple peptide patterns of bacterial, fungal or oomycete origin (Boutrot & Zipfel, 2017; Ranf, 2017).

LRR‐RPs and LRR‐RKs consist of an extracellular LRR‐domain for ligand binding and a single‐pass transmembrane domain for plasma‐membrane localization. LRR‐RKs possess an intracellular protein kinase domain that is lacking in LRR‐RPs. Instead, LRR‐RPs constitutively interact with adaptor kinases such as SOBIR1 (SUPPRESSOR OF BAK1‐INTERACTING RECEPTOR‐LIKE KINASE) to form a bipartite receptor kinase complex (Gust & Felix, 2014; Liebrand *et al*., 2014; Domazakis *et al*., 2018). Upon ligand binding, both receptor types recruit SERK (SOMATIC EMBRYOGENESIS RECEPTOR KINASE) co‐receptors, such as BAK1/SERK3 (BRI1 (BRASSINOSTEROID INSENSITIVE 1)‐ASSOCIATED RECEPTOR KINASE 1) for intracellular signal transduction (Chinchilla *et al*., 2007; Heese *et al*., 2007; Albert *et al*., 2015; Postma *et al*., 2016).

Arabidopsis LRR‐RK FLS2 (FLAGELLIN SENSING 2) recognizes bacterial flagellin or a peptide thereof (flg22) (Gomez‐Gomez & Boller, 2000) and undergoes rapid complex formation with BAK1 (Chinchilla *et al*., 2007; Heese *et al*., 2007; Roux *et al*., 2011). 3D‐Structure elucidation of the FLS2 BAK1 ectodomain complex revealed that the ligand acts as molecular glue to facilitate receptor/co‐receptor interaction (Sun *et al*., 2013). Arabidopsis LRR‐RP RLP23 recognizes an immunogenic peptide motif (nlp20) found within necrosis and ethylene‐inducing peptide 1 (NEP1)‐like proteins (NLP) (Albert *et al*., 2015). NLPs are produced by numerous plant pathogenic bacteria, oomycetes and fungi (Ottmann *et al*., 2009; Lenarčič *et al*., 2017), which makes the nlp20 pattern unique by occurring in three different microbial lineages (Böhm *et al*., 2014b). Like FLS2, functionality of LRR‐RP/SOBIR1 complexes also involves ligand‐induced recruitment of BAK1 or related SERKs (Albert *et al*., 2015; Postma *et al*., 2016), but little information about the molecular architecture of such a tripartite complex is available. Gel filtration experiments conducted with recombinant RLP23 and BAK1 LRR ectodomains in the absence or presence of nlp20 suggest that nlp20 mediates RLP23 BAK1 interaction and that SOBIR1 is not required for this interaction (Albert *et al*., 2015).

Ligand‐induced PRR complex formation suggests suppression of this process in the absence of the ligand. Indeed, LRR‐RKs BIR2 (BAK1‐INTERACTING RECEPTOR‐LIKE KINASE 2) and BIR3 have been shown to bind to BAK1 in the resting state and to prevent complex formation with LRR‐RK PRRs such as FLS2 (Halter *et al*., 2014; Imkampe *et al*., 2017). BIR2 and BIR3 are considered negative regulators of PTI responses mediated through activation of LRR‐RK‐type PRRs (Saijo *et al*., 2018).

BAK1 and FLS2 both bind to BIK1 (BOTRYTIS‐INDUCED KINASE 1), a receptor‐like cytoplasmic kinase (RLCK) (Lu *et al*., 2010; Zhang *et al*., 2010). Upon elicitation, FLS2 BAK1 heteromeric complexes are formed and BIR2 BAK1 and BIR3 BAK1 complexes dissociate (Halter *et al*., 2014; Imkampe *et al*., 2017). BIK1 phosphorylates both FLS2 and BAK1 and is itself phosphorylated by BAK1 (Lu *et al*., 2010; Zhang *et al*., 2010). Activated BIK1 then phosphorylates the NADPH oxidase RBOHD (RESPIRATORY BURST OXIDASE HOMOLOG PROTEIN D) (Kadota *et al*., 2014; Li *et al*., 2014) and, likely, other substrates. Whether a similar scenario underlies PTI activation through LRR‐RP‐type PRRs remains unknown.

Due to the similar architecture of LRR‐RK and LRR‐RP PRRs and due to recruitment of BAK1 (and related SERKs) by both PRR types it was suggested that these proteins would function in a virtually identical manner and that LRR‐RP/SOBIR1 complexes constitute heterodimeric LRR‐RKs (Gust & Felix, 2014). This view is supported by both receptor complexes mediating plant resistance to microbial infections (Zipfel *et al*., 2004; Albert *et al*., 2015). To test this hypothesis, we have conducted a comprehensive survey of PTI‐associated responses mediated through either an LRR‐RK (FLS2) or LRR‐RP (RLP23)‐type PRR. Choice of this pair of PRRs was dictated by the fact that for both receptors similarly sized, pure peptide ligands are available. We found both qualitative and quantitative differences in immunity‐associated responses triggered by these patterns as well as an unforeseen differential role of BIK1 in LRR‐RK and LRR‐RP‐mediated PTI. Similar results were obtained for LRR‐RK EFR (ELONGATION FACTOR THERMO UNSTABLE RECEPTOR) and LRR‐RP RLP42 suggesting that, despite very similar receptor complex architectures, cellular outputs mediated through either receptor type are distinct and differ in their requirement for BIK1.

## Materials and Methods

### Plant material

All plant lines and mutants used in this study are in *Arabidopsis thaliana* accession Columbia‐0 (Col‐0) background (listed in Supporting Information Table S1).

### Reactive oxygen species (ROS) and ethylene measurement

The detection of ROS and ethylene in leaf pieces of 5‐wk‐old Arabidopsis plants was performed as described (Felix *et al*., 1999; Albert *et al*., 2010). Leaves were cut into pieces and floated on H_2_O overnight before measurement. For ROS assays, one leaf piece per well was placed in a 96‐well plate containing 20 μM L‐012 (Wako Pure Chemical Industries Ltd, Osaka, Japan) and 2 μg ml^−1^ peroxidase. Luminescence was measured both before (background) and over 1 h following elicitation or mock treatment using a Mithras LB 940 luminometer (Berthold Technologies, Bad Wildbad, Germany). For ethylene measurement, four randomly selected leaf pieces were incubated in a 6.5 ml glass tube with 0.5 ml 20 mM MES buffer, pH 5.6 and the indicated elicitor. At least three replicates were performed for all treatments. Ethylene accumulation was measured by gas chromatographic analysis (GC‐14A; Shimadzu, Duisburg, Germany) of 1 ml of the air drawn from the closed tube with a syringe after the indicated incubation time.

### Salicylic acid and camalexin measurement

Here, 5‐wk‐old Arabidopsis leaves were infiltrated with peptide solution or ddH_2_O. For analysis of salicylic acid (SA) and camalexin, 200 mg of fresh plant leaves were harvested and homogenized in liquid nitrogen. Extraction of the free analytes was carried out with 1.5 ml ethyl acetate, containing 0.1% (v/v) formic acid and the internal standards 3‐hydroxybenzoeic acid and dihydro‐jasmonic acid. Samples were incubated at 28°C for 60 min after a 10 min sonification step in an ultrasonic bath. After centrifugation at 18 500 ***g***, 1.2 ml supernatant was transferred into a new tube. The ethyl acetate was removed in a vacuum concentrator. Derivatization was performed with a 1 : 1 mixture of 70 μl TMSDM (2.0 M in diethyl ether) : methanol for 20 min at 25°C. Determination of the analytes in 1 μl injected volume was performed by GC/MS (Agilent 6890 GC and Agilent 5973 single quad mass spectrometer, Agilent Technologies, Santa Clara, CA, USA), using split injection mode and a SPB‐50 column (30 m, 0.25 mm internal diameter, Supelco, Sigma–Aldrich, Munich, Germany). The GC oven temperature was held at 70°C for 5 min, then ramped at 15°C min^−1^ to 270°C, then ramped at 75°C min^−1^ to 280°C and afterwards held for an additional 10 min at 280°C. Helium was used as carrier gas with a flow rate of 1 ml min^−1^. The mass spectrometer was operated in electron impact ionization (EI) and selected ion monitoring (SIM) mode.

### RNA‐sequencing and data analysis

For this process, 10‐d‐old Arabidopsis seedlings grown on half‐strength MS medium under short‐day conditions were moved from plates to water 1 d before treatment with H_2_O, 0.5 μM flg22 or nlp20 for 0, 1, 6 and 24 h. Total RNA was extracted using the RNeasy Plant Mini Kit (Qiagen, Hilden, Germany), and DNA contamination was removed using a RNase‐free DNase Set (Qiagen). RNA quantity and quality were checked using an ABI 3730xl DNA Analyzer (Applied Biosystems, Foster City, CA, USA) and a Qubit^®^ 2.0 Fluorometer (Invitrogen, Carlsbad, CA, USA). The cDNA library was prepared from 2 μg RNA using Illumina^®^ TruSeq^®^ RNA Sample Preparation Kits (Illumina, San Diego, CA, USA).

The cDNA library was sequenced using a HiSeq2000 with cBot instrument (Illumina). RNA‐seq reads were aligned to CD‐HIT (Li & Godzik, 2006) optimized TAIR10 Col‐0 reference transcripts using bowtie 2 (Langmead *et al*., 2009). Uniquely mapped reads were quantified per representative gene model using eXpress (Roberts & Pachter, 2013). Only transcripts with counts per million ≥ 2 in at least two replicates were used for differential expression analysis using edgeR (Robinson *et al*., 2010; McCarthy *et al*., 2012). This package internally estimates size factors for each sample, calculates dispersion for each gene, and then fits a negative binomial generalized linear model to detect differentially expressed genes considering the size factors and dispersion values.

### Quantitative real‐time PCR

Total RNA was isolated from leaves of 5‐ to 6‐wk‐old Arabidopsis plants using the NucleoSpin^®^ RNA Plus kit (Macherey‐Nagel, Düren, Germany). First‐strand cDNA synthesis was performed from 1 μg of total RNA using RevertAid™ MuLV reverse transcriptase (Thermo Scientific, Waltham, MA, USA). Quantitative PCR reactions and measurements were performed with the iQ5 Multi‐color real‐time PCR detection system (Bio‐Rad, Hercules, CA, USA) using the SYBR Green Fluorescein Mix (Thermo Scientific) and gene‐specific primers for *PAD3* (Primers PAD3_Forward 5′‐CGAGCATCTTAAGCCTGGAA‐3′ and PAD3_Reverse 5′‐ACTCCACCAATCCCTGCTAC‐3′), *CYP71A13* (Primers CYP71A13_Forward 5′‐TCGGTTGCATCCTTCTCTTC‐3′ and CYP71A13_Reverse 5′‐GTCCCCATATCGCAGTGTCT‐3′), *MLO12* (Primers MLO12_Forward 5′‐ACGGTGGTTGTCGGTATAAGCC‐3′ and MLO12_Reverse 5′‐AGGGCAGCCAAAGATATGAGTCC‐3′) and PR1 (qPR1_F 5′‐CGCTGCGAACACGTGCAATG‐3′ and qPR1_R 5′‐CCACGAGGATCATAGTTGCAAC‐3′). Transcript levels of target genes were normalized to the transcript levels of the house keeping gene *EF‐1α* (Primers EF‐1a_Forward 5′‐GAGGCAGACTGTTGCAGTCG‐3′ and EF‐1a_Reverse 5′‐TCACTTCGCACCCTTCTTGA‐3′) as a reference gene, and calibrated to the levels of mock infiltration in wild‐type plant (set as 1), according to the 2^−∆∆Ct^ (cycle threshold) method (Livak & Schmittgen, 2001). Data are presented as the average of three replicates ± SD, and three independent experiments were performed.

More information on methods used here can be found in Methods S1.

## Results

### Similar but distinct immune responses are induced by both, nlp20 and flg22

Microbe‐derived immunogenic structures trigger largely overlapping plant response patterns (Bigeard *et al*., 2015) suggesting that a generic, rather than a microbial pattern‐specific, response underlies the plant's ability to restrict infections. To investigate plant responses mediated through the activation of LRR‐RK and LRR‐RP‐type PRRs we have chosen a systematic approach to compare flg22/FLS2‐ and nlp20/RLP23‐mediated early and late plant defenses. Towards this end, we have used highly purified synthetic peptide preparations, the same plant seed stocks and identical experimental setups. All experiments were conducted with both elicitors in parallel, and samples were handled in a randomized way. Such stringent conditions were employed because small variations in plant growth and handling conditions have previously been reported to cause rather large differences in plant phenotypes (Massonnet *et al*., 2010).

**Figure 1:**
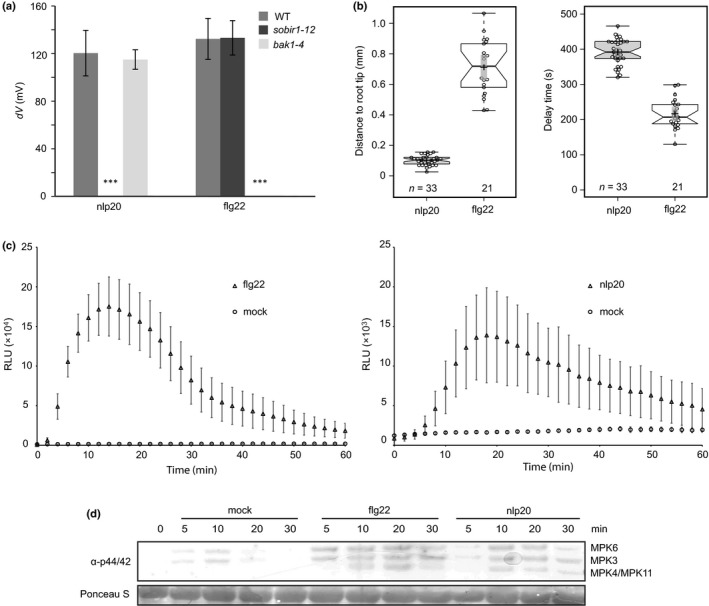
Comparison of early cellular responses after flg22 and nlp20 treatment. (a) Leaves of Arabidopsis Col‐0 wild‐type (WT) plants or the indicated mutants were treated with 100 nM nlp20 or 10 nM flg22, and changes in membrane potential were monitored continuously. Mean values ± SD (11 ≤ *n *≤* *15) of the difference in membrane potential (dV) after 2 min treatment are shown compared with the untreated control. Asterisks indicate significant differences to wild‐type Arabidopsis as determined by Student's *t*‐test: ***, *P *<* *0.001. (b) Roots of the *R‐GECO1*‐transgenic Arabidopsis line were treated with 10 μM nlp20 or flg22 and either the distance between the root tip and the first detectable calcium signal (left panel) or the delay time between elicitor application and the first detectable calcium signal (right panel) were quantified. Boxplots show individual data points as circles (*n*(nlp20) = 33; *n*(flg22) = 21), median values as center lines, mean values as crosses and 95% confidence intervals of the means as gray transparent rectangles. (c) Arabidopsis leaf disks were treated with 0.5 μM flg22 or nlp20, or water as the control (mock), and reactive oxygen species (ROS) production was monitored over time. Bars present means ± SD (*n *≥* *6) of relative fluorescence units (RLU). (d) Arabidopsis leaves were infiltrated with 1 μM flg22 or nlp20 or water as control and harvested at the indicated time points. The activation of the mitogen‐activated protein kinases (MAPKs) was visualized by western blot analysis using the phospho‐p44/42 MAP kinase antibody. Ponceau S Red‐staining of the membrane served as a loading control. All experiments were performed three times with similar results.

Plasma membrane depolarization is among the very first responses observed in elicited plants (Jeworutzki *et al*., 2010). Treatment with either elicitor resulted in very rapid and substantial changes in the plasma membrane potential, which is dependent on co‐receptors such as BAK1 and SOBIR1 (Fig. 1a). However, neither response timing nor amplitude differed significantly in flg22‐ or nlp20‐treated plants (Fig. S1).

Changes in cytoplasmic calcium concentrations are also among the earliest changes observed in elicited plant cells. To determine spatiotemporal differences between the calcium signal elicited by flg22 and nlp20, we treated roots of seedlings expressing the genetically encoded calcium sensor R‐GECO1 (Keinath *et al*., 2015) with flg22, nlp20 or medium lacking either elicitor. Upon treatment with flg22 we observed a response mostly in the elongation zone with a median delay of *c*. 3 : 27 min (Figs 1b, S2; Video S1), which is consistent with previously reported Ca^2+^ signatures elicited by flg22 (Keinath *et al*., 2015). By contrast, treatment with nlp20 elevated calcium levels that were detected exclusively in the meristematic zone of the root with a median delay of *c*. 6 : 32 min (Figs 1b, S2, Video S2). Therefore, both patterns triggered increases in cytoplasmic Ca^2+^ levels, but with different timing and location.

Treatment with both patterns resulted in the production of ROS as well as in the phosphorylation of mitogen‐activated protein kinases (MAPKs). However, flg22‐induced plant responses were faster and stronger than those evoked upon nlp20‐treatment for these two outputs. For example, ROS production was detectable nearly without lag phase in flg22‐treated Arabidopsis leaf disks, whereas ROS levels increased after *c*. 6 min upon elicitation in nlp20‐treated leaf tissue. In addition, amplitudes varied significantly between the two treatments. Maximum ROS levels were reproducibly higher upon flg22‐treatment by approximately one order of magnitude, and neither amplitude nor response‐onset were changed by increasing elicitor concentrations (Figs 1c, S3a). Moreover, ROS levels declined much faster in flg22‐treated leaves, reaching 47% and 10% of the maximum levels at 30 and 60 min upon elicitation, respectively. In nlp20‐treated leaves this decline was delayed significantly (75% and 33% after 30 and 60 min, respectively). MAPK activation was detectable within 5 min or 10 min upon elicitation with flg22 or nlp20, respectively, and, irrespective of the concentrations used, was stronger in flg22‐treated samples compared with nlp20 application (Figs 1d, S3b).

Differences in FLS2‐ vs RLP23‐mediated immune activation may represent a peculiar feature of the tested patterns or may rather be representative for immune activation through the respective LRR‐RK and LRR‐RP families. To test this, we also conducted a series of experiments with two additional molecularly defined proteinogenic patterns: fungal polygalacturonase PG3, which is recognized by LRR‐RP RLP42 (Zhang *et al*., 2014), and elf18, a bacterial pattern recognized by the LRR‐RK, EFR (Zipfel *et al*., 2006). Consistent with the results obtained with flg22 and nlp20, elf18 induced stronger and faster ROS production than PG3 (Fig. S4a) which was also reported for the stimulation of yet another RP, RLP30 by a partially purified, proteinaceous *Sclerotinia sclerotiorum* extract (Zhang *et al*., 2013).

### Nlp20 and flg22 trigger largely overlapping transcriptional reprogramming

**Figure 2:**
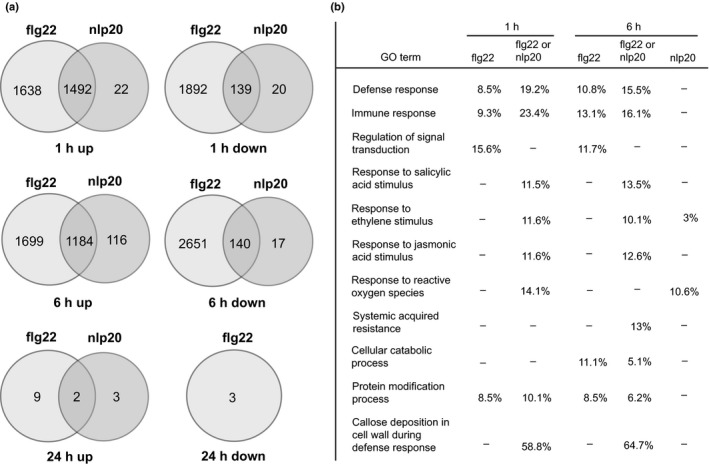
Flg22 and nlp20 induce overlapping transcriptional changes. (a) Arabidopsis wild‐type seedlings were treated with water or 0.5 μM nlp20 or flg22 for 1, 6 and 24 h, and isolated RNA was subjected to RNA sequencing. Shown are the numbers of up‐ or downregulated transcripts, with a false discovery rate (FDR) ≤ 0.01 and a fold change ≥ 2. (b) Selected gene ontology (GO) terms of upregulated transcripts. The percentages indicate the number of upregulated genes with the GO term that were identified in the sample relative to the total number of genes with the GO term. For FDR > 0.05, no percentage is shown, as well as for the 1 h nlp20 treatment, which did not yield any GO terms. ‘flg22 or nlp20’ indicates overlapping genes.

To monitor changes in overall gene expression patterns in elicited plants we conducted RNA sequencing (RNA‐seq) experiments. Arabidopsis seedlings were treated with either water (mock treatment), 0.5 μM flg22 or 0.5 μM nlp20 for 1, 6 or 24 h. Marker gene expression was tested by qRT‐PCR and three replicates were further subjected to RNA sequencing. Genes differentially expressed (false discovery rate (FDR) ≤ 0.01 and fold change ≥ 2) were defined using the edgeR software package (Robinson *et al*., 2010; McCarthy *et al*., 2012). After treatment for 1 or 6 h, flg22 caused a dramatic transcriptome reprogramming. At 1 h after treatment, 3130 genes were upregulated, and 2031 genes were downregulated (Fig. 2a). Nlp20 treatment also caused transcriptional upregulation of a large number of genes, but to a lesser extent than flg22 treatment. Unlike flg22, only a few genes showed reduced expression upon nlp20 treatment (Fig. 2a). Ninety‐seven percent (1 h treatment) and 91% (6 h treatment) of transcripts that changed after nlp20 treatment were similarly affected by flg22 application. Conversely, only 32% (1 h treatment) and 23% (6 h treatment) of transcripts that changed after flg22 treatment were similarly affected by nlp20 application. Although we could observe a massive transcriptional reprogramming at early time points, only a few genes showed altered expression in response to either pattern after 24 h (Fig. 2a). Altogether, our findings suggested that nlp20‐induced genes represent a fraction of flg22‐induced genes.

Singular enrichment analysis was performed using agriGO (Du *et al*., 2010) to identify the enrichment of Gene ontology (GO) terms using an FDR of ≤ 0.01 (Table S2). Both flg22 and nlp20 triggered the expression of hormone‐responsive genes and many immunity‐related genes (Fig. 2b). Genes related to late defense responses, such as systemic acquired resistance or callose deposition, were predominantly found among the upregulated transcripts of the 6 h treatments with flg22 or nlp20 compared to the 1 h treatments. Interestingly, a subgroup of genes only responded to flg22 (Fig. 2a; Table S3). These genes are predicted to be involved in immunity, regulation of signal transduction and other molecular processes (Fig. 2b; Table S3). Moreover, many genes related to metabolic processes were regulated specifically after flg22 treatment. A smaller subset of genes was specifically induced following nlp20‐treatment (Fig. 2a; Table S3). We observed an enrichment of GO terms related to the ROS response 6 h after nlp20‐treament, but not after flg22 treatment, suggesting that flg22 and nlp20 have differential prolonged effects on the oxidative status in the plant (Table S3). In summary, both flg22 and nlp20 treatments cause massive transcriptional reprograming. Notably, whereas most genes responsive to nlp20 are also up‐ or downregulated in flg22 samples, flg22 treatment causes differential regulation of an additional specific set of genes. As both patterns prime plants for immunity to subsequent infection (Zipfel *et al*., 2004; Albert *et al*., 2015), it is tempting to speculate that nlp20‐induced genes comprise a minimum gene set that is sufficient for immune activation in Arabidopsis. Notably, most of the genes expressed in a flg22‐specific manner were found not to be induced upon nlp20 treatment even when a lower induction threshold (> 1.7) was considered. Furthermore, increasing the nlp20 concentration by 10‐fold did not result in an induction of, for instance, the flg22‐inducible gene *MLO12* (*MILDEW RESISTANCE LOCUS O 12*) (Fig. S3c).

### Nlp20 differentially regulates phytohormone and phytoalexin production

**Figure 3:**
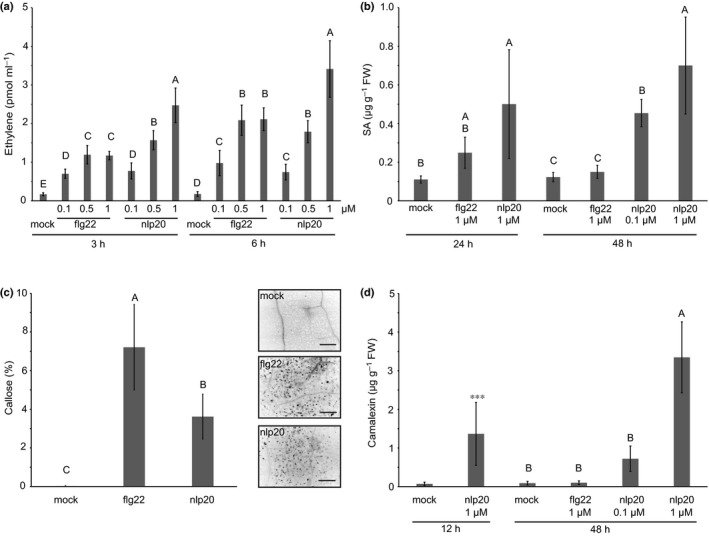
Comparison of late cellular responses after flg22 and nlp20 treatment. (a) Ethylene accumulation after treatment with water (mock), flg22 or nlp20 at indicated concentrations, measured after 3 and 6 h. Bars present average values ± SD (*n *≥* *3). Within one time point, different letters mean significant differences (*P *<* *0.05) using ANOVA followed by Student's *t*‐test for all possible individual comparisons. (b) Salicylic acid (SA) levels in Arabidopsis leaves after 24 and 48 h of treatment with 1 μM flg22, 1 μM nlp20 (also 0.1 μM after 48 h), or water (mock). Bars (μg SA g^−1^ FW) represent means ± SD of four replicates. Different letters indicate significant differences (*P *<* *0.05) using ANOVA followed by Student's *t*‐test for all possible individual comparisons. (c) Callose production in Arabidopsis leaves infiltrated with 1 μM flg22 or nlp20, or water (mock). Callose deposition was visualized with aniline blue 16 h after infiltration and evaluated using a fluorescence microscopy. Details of leaf samples 10‐times magnified under UV light are shown on the right, dark spots indicate callose deposition; bars, 25 μm. The diagram on the left depicts callose deposition in percentage ± SD of three biological replicates, counted as dark pixels. (d) Camalexin levels were determined in leaves infiltrated with 1 μM flg22 or nlp20 (also 0.1 μM for 48 h), or water (mock) and harvested after 12 or 48 h. Bars (μg camalexin g^−1^ FW) present average values ± SD (*n *=* *4). Different letters indicate significant differences (*P *<* *0.05) using ANOVA followed by Student's *t*‐test for all possible individual comparisons. Asterisks indicate significant differences to the water control treatment as determined by Student's *t*‐test: ***, *P *<* *0.001.

Plant hormones SA and ethylene have been implicated in plant immunity (Pieterse *et al*., 2012). Leaves treated with either RK or RP ligands produced the stress hormone ethylene. However, both nlp20 and PG3 induced more ethylene production than flg22 and elf18 when tested at higher concentrations (Figs 3a, S4b). SA levels increased within 24 h of pattern treatment and were generally higher in nlp20‐treated plants than in plants treated with flg22 (Fig. 3b). This difference became even more pronounced 48 h post elicitation, where SA levels remained high in nlp20‐treated samples but not in flg22‐treated samples. Likewise, expression of the SA‐marker gene *PR1* (*PATHOGENESIS‐RELATED 1*) was more pronounced in nlp20‐ and PG3‐treated leaves compared with samples after flg22 or elf18 infiltration (Fig. S4c).

Callose deposition is another hallmark of PTI (Ellinger & Voigt, 2014). Both patterns triggered the production of this protective carbohydrate, albeit to a significantly lower level in nlp20‐ and PG3‐treated leaves compared with flg22 and elf18 treated (Figs 3c, S4d), confirming and extending recent findings (Albert *et al*., 2015). Camalexin is a Brassicaceae indole alkaloid phytoalexin that is produced upon microbial infection or pattern treatment and is an effective deterrent of bacterial and fungal pathogens (Glawischnig, 2007). As shown in Fig. 3d, nlp20 treatment, but not flg22 treatment, resulted in the massive production of camalexin marking yet another striking difference in nlp20‐induced vs flg22‐induced plant responses. Consistently, compared with flg22 and elf18 stimulation, a treatment with PG3 (Fig. S4e) and the RLP30‐ligand SCFE1 (*SCLEROTINIA CULTURE FILTRATE ELICITOR 1*; Zhang *et al*., 2013) resulted in more enhanced transcript accumulation of camalexin biosynthesis genes *PAD3* (*PHYTOALEXIN DEFICIENT 3*) and *CYP71A13* (*CYTOCHROME P450 MONOOXYGENASE 71A13*).

### Role of BAK1 phosphorylation sites is shared between flg22‐ and nlp20‐induced immune responses

**Figure 4:**
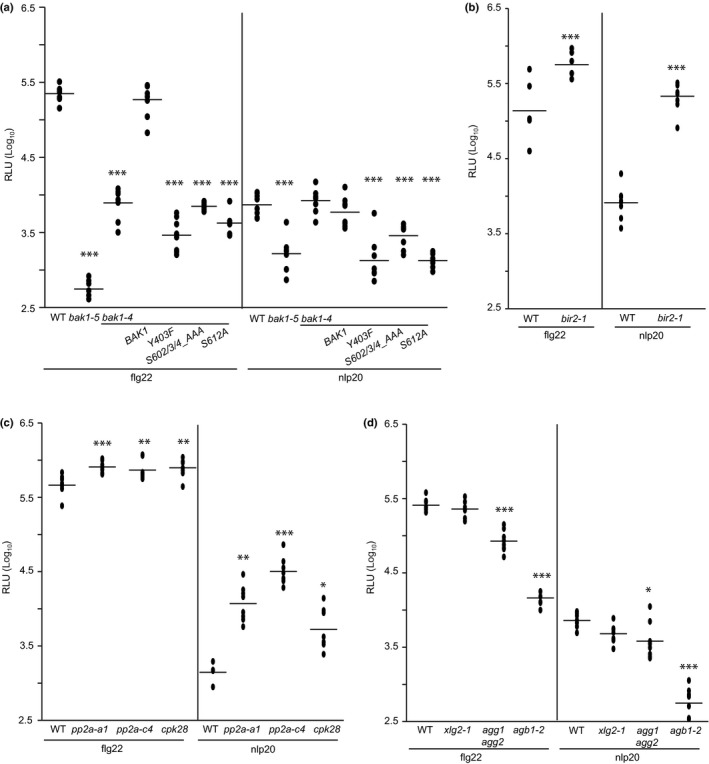
Signaling components playing similar roles in nlp20‐ and flg22‐induced immunity. (a) Leaf disks of Arabidopsis wild‐type (WT) plants or the mutant lines *bak1‐4*,* bak1‐5*,* bak1‐4*/*BAK1*,* bak1‐4/BAK1_Y403F, bak1‐4/BAK1_S602/3/4_AAA* and *bak1‐4/BAK1_S612A* were treated with 0.5 μM flg22 or nlp20, respectively, and reactive oxygen species (ROS) accumulation was determined. (b–d) ROS production was compared in WT Arabidopsis plants vs the mutant lines (b) *bir2‐1*, (c) *pp2a‐a1*,* pp2a‐c4*, and *cpk28*, or (d) *xlg2‐1, agg1 agg2 and agb1‐2*, each treated with either 0.5 μM flg22 or nlp20. All presented data (relative fluorescence units, RLU) show peak values minus background values as dots (*n *≥* *6) and the means as lines. Asterisks indicate significant differences compared with the wild‐type as determined by Student's *t*‐test: *, *P *<* *0.05; **, *P *<* *0.01; ***, *P *<* *0.001. The experiments were performed three times with similar results.

Our side‐by‐side analysis of FLS2‐ and RLP23‐mediated immunity revealed that these receptors trigger overlapping but distinct immune responses. We next sought to identify signaling components that play shared or divergent roles in RP‐ and RK‐mediated immunity. To that end, we tested mutants with defects in FLS2‐signaling genes to determine whether these genes play similar roles in RLP23‐mediated immunity. BAK1 acts as a ligand‐binding co‐receptor for both FLS2 and RLP23 (Sun *et al*., 2013; Albert *et al*., 2015). Pattern treatment leads to recruitment into heteromeric complexes with both PRR types, and BAK1 and related SERK family members have been shown to be required for PTI activation (Ma *et al*., 2016). Several phosphorylation sites in the BAK1 kinase domain have been identified as important for BAK1‐mediated PTI (Perraki *et al*., 2018). We have used mutants *bak1‐4*/*BAK1_Y403F*,* bak1‐4*/*BAK1_S602AIS603A/S604A*, and *bak1‐4*/*BAK1_S612A* to analyze the importance of these phosphorylation sites for the flg22‐ and nlp20‐induced ROS burst. As shown in Fig. 4(a), flg22‐induced ROS burst was strongly reduced in all three mutant genotypes and resembled that observed in *bak1‐4* mutants. This finding confirms that residues Y403, S602/3/4 and S612 are important for flg22 sensitivity in Arabidopsis (Perraki *et al*., 2018). Upon treatment with nlp20, the *bak1‐4* mutant mounted ROS levels that were similar to those in wild‐type plants, this result is in contrast with reduced ROS levels observed upon flg22 treatment in this mutant (Figs 4a, S5). This finding suggests that BAK1 and related members of the SERK protein family play different roles in flg22‐ and nlp20‐mediated immune signaling. Importantly, ROS levels in *bak1‐4* mutants that complemented the previously mentioned phosphorylation site mutants were strongly reduced upon nlp20 treatment, suggesting that these mutations are (semi)dominant. Strongly reduced ROS levels observed in pattern‐treated *bak1‐5* plants showed that this dominant negative mutation (Schwessinger *et al*., 2011) affected RLP23‐ and FLS2‐mediated PTI in the same way (Fig. 4a).

### Negative regulators of flg22 signaling are involved in nlp20‐triggered immunity

Three independent negative regulatory mechanisms prevent the activation of FLS2 in the absence of its cognate ligand, bacterial flagellin. LRR‐RK BIR2 interacts with BAK1 in the noninduced state thereby preventing BAK1 FLS2 complex formation (Halter *et al*., 2014). Similarly, protein Ser/Thr phosphatase type 2A constitutively interacts with BAK1 and controls its phosphorylation status (Segonzac *et al*., 2014). FLS2 activation disrupts this negative control circuit, resulting in BAK1 phosphorylation and activation of PTI. Furthermore, CALCIUM‐DEPENDENT PROTEIN KINASE 28 (CPK28) controls the stability and phosphorylation status of BIK1, an RLCK and important hub in LRR‐RK‐mediated PTI (Monaghan *et al*., 2015; Couto *et al*., 2016; Wang *et al*., 2018). *bir2*,* pp2A* and *cpk28* loss‐of‐function alleles were tested for changes in flg22‐ or nlp20‐induced ROS production. All genotypes showed higher ROS levels after treatment with either pattern compared with the ROS levels observed in elicited wild‐type plants (Fig. 4b,c). These findings suggest that certain negative regulatory control mechanisms are the same for both LRR‐RK and LRR‐RP‐type PRRs.

Heterotrimeric G proteins positively regulate flg22‐dependent PTI through direct interaction with FLS2 and BIK1 (Liang *et al*., 2016; Wang *et al*., 2018). As shown in Fig. 4(d), mutants depleted of Gβ protein AGB1 (ARABIDOPSIS GTP BINDING PROTEIN BETA 1) and Gγ subunits AGG1 (ARABIDOPSIS G PROTEIN GAMMA‐SUBUNIT 1) and AGG2, respectively, mounted less ROS in response to flg22 or nlp20 treatment than wild‐type plants, whereas genetic inactivation of the noncanonical Gα subunit *XLG2* (*EXTRA‐LARGE GTP‐BINDING PROTEIN 2*) did not significantly affect pattern‐triggered ROS production in our hands.

### BIK1 has opposite roles in LRR‐RP‐ and LRR‐RK‐mediated immune signaling

**Figure 5:**
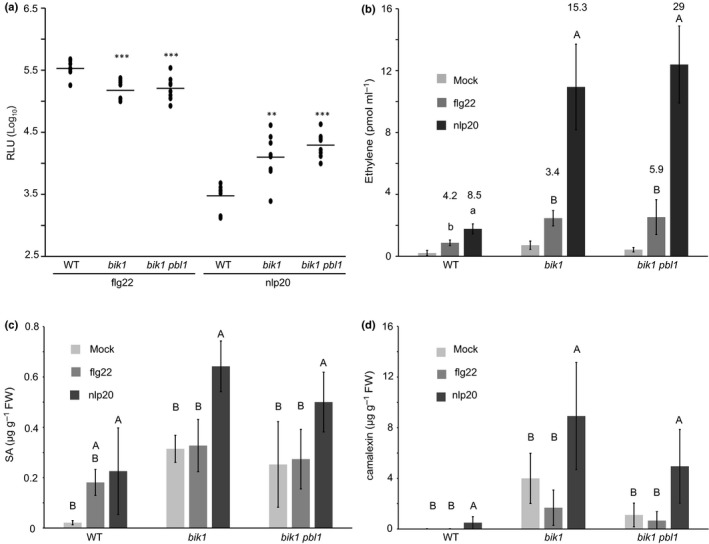
BIK1 plays a differential role in flg22 and nlp20 triggered signaling. (a, b) Leaf disks of Arabidopsis wild‐type (WT) plants and *bik1* or *bik1 pbl1* mutants were treated with 0.5 μM flg22 or nlp20, respectively. (a) For reactive oxygen species (ROS) accumulation (relative fluorescence units, RLU), peak value minus background value are shown as dots (*n *≥* *6) and means are presented as lines. Asterisks indicate significant differences to the wild‐type control treatment as determined by Student's *t*‐test: **, *P *<* *0.01; ***, *P *<* *0.001. (b) For ethylene production, bars represent mean ethylene production ± SD of four replicates after 6 h of treatment. Within one line, letters indicate significant differences (*P *<* *0.05) and within one treatment, different cases indicate significant differences (*P *<* *0.05) as determined by ANOVA followed by Student's *t*‐test for all possible individual comparisons. Numbers indicate the fold changes between elicitor and mock treatment in the corresponding line. (c, d) Mature leaves of Arabidopsis WT plants or mutants were infiltrated with 0.5 μM flg22 or nlp20, respectively, or water as control (mock) and harvested after 24 h for determination of (c) salicylic acid (SA) or (d) camalexin accumulation. Bars (μg g^−1^ FW) present means ± SD of four biological replicates. Within one line, different letters indicate significant differences (*P *<* *0.05) as determined by ANOVA followed by Student's *t*‐test for all possible individual comparisons.

RLCK BIK1 and related PBL1 (AVRPPHB SUSCEPTIBLE 1‐LIKE 1) are positive regulators of flg22‐mediated PTI (Lu *et al*., 2010; Zhang *et al*., 2010). We therefore tested *bik1* and *bik1 pbl1* mutants for flg22‐ and nlp20‐induced ROS production. Surprisingly, while we could confirm reduced ROS levels after flg22 treatment relative to those in wild‐type plants (Zhang *et al*., 2010), we found substantially increased ROS levels in both mutant genotypes after nlp20 challenge (Figs 5a, S5). We further investigated SA, ethylene and camalexin levels in *bik1* or *bik1 pbl1* mutant lines. As shown in Fig. 5(b–d), *bik1* and *bik1 pbl1* mutants generally had higher levels of phytohormones, particularly SA, and camalexin. Nevertheless, upon pattern treatment ethylene production was more strongly induced in *bik1* and *bik1 pbl1* mutants in nlp20 samples compared with flg22 samples. For instance, *bik1* plants accumulated 15.3‐fold more ethylene after nlp20 treatment and 3.4‐fold more ethylene after flg22 challenge when compared with mock controls (Fig. 5b). Notably, SA levels increased in flg22‐treated wild‐type plants, but not in *bik1* and *bik1 pbl1* genotypes (Figs 3b, 5c). Likewise, these mutants also did not show camalexin accumulation after flg22 challenge (Fig. 5d). By contrast, *bik1* and *bik1 pbl1* mutants displayed increased levels of SA and camalexin following nlp20 treatment in comparison with the wild‐type control. These findings suggested a novel role for BIK1 as a negative regulator of RLP23‐mediated immunity, by contrast with its positive regulatory role in FLS2‐mediated immunity.

**Figure 6:**
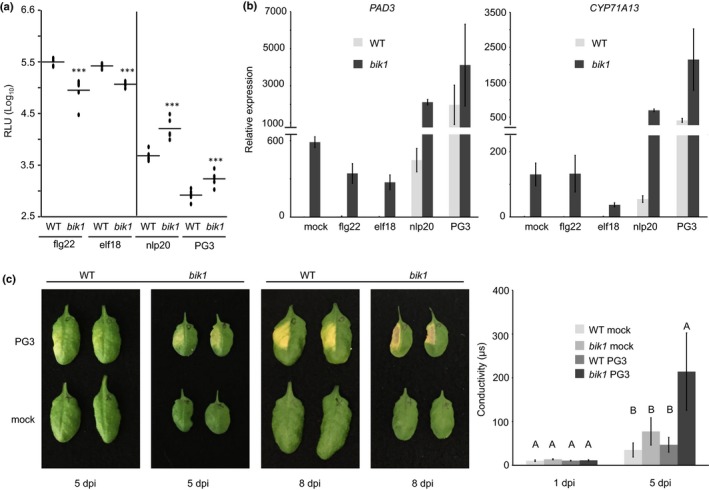
BIK1 plays a differential role in LRR‐RP and LRR‐RK signaling. (a) Arabidopsis leaf discs of wild‐type (WT) or *bik1* mutant plants were treated with each 0.5 μM flg22, elf18, nlp20, or 0.1 μM PG3 and reactive oxygen species (ROS) accumulation (relative fluorescence units, RLU) was determined. Peak value minus background value are shown as dots (*n *≥* *6) and means are presented as lines. Asterisks indicate significant differences compared with the wild‐type control treatment as determined by Student's *t*‐test: ***, *P *<* *0.001. (b) Transcriptional profiling of camalexin biosynthesis genes by quantitative real‐time PCR (qRT‐PCR). Leaves of Arabidopsis wild‐type or *bik1* mutant plants were infiltrated with water (mock), each 0.5 μM flg22, elf18, nlp20, or PG3 and collected 6 h after treatment. Relative expression of the indicated genes was normalized to the levels of the *EF‐1α* transcript and calibrated to the levels of the mock treatment in the WT control. Data present means of three technical replicates ± SD. (c) Leaves of Arabidopsis WT or *bik1* mutant plants were infiltrated with water (mock) or 0.5 μM PG3 and visible cell death symptoms were monitored at 5 and 8 d post infiltration (dpi) (left panel). In addition, cell death progression 1 and 5 dpi was monitored by ion leakage assays using excised leaf discs floating on water (right panel). Bars represent mean conductivity ± SD (*n *=* *4), capital letters indicate significant differences (*P *<* *0.001) as determined by Student's *t*‐test. Each experiment was performed three times with similar results.

Notably, in *bik1* mutants higher ROS levels were observed also after PG3 treatment in comparison with those found in wild‐type plants as opposed to lower levels observed after treatment with flg22 and elf18 (Fig. 6a). Furthermore, camalexin biosynthesis genes, *CYP71A13* and *PAD3*, were more strongly transcriptionally up‐regulated in *bik1* mutants upon challenge with LRR‐RP ligands nlp20 and PG3 compared with flg22 or elf18 treatment (Fig. 6b).

Unlike other known RP elicitors, PG3 is an inducer of plant hypersensitive cell death (Zhang *et al*., 2014). We therefore analyzed cell death development in wild‐type plants and *bik1* mutants infiltrated with PG3. Again, we observed strongly enhanced phenotypes in *bik1* leaves relative to those in the wild‐type control. Ion conductivity measurements in leaf discs revealed significant differences between wild‐type plants and *bik1* mutants after PG3 treatment, confirming the relatively more severe, cell death in *BIK1*‐deficient plants (Fig. 6c). In summary, *bik1* (and *bik1 pbl1*) mutants, despite having higher basal phytohormone and camalexin levels, reacted more strongly to nlp20 (and PG3) than to flg22 (and elf18) treatment, indicating that BIK1 is differentially involved in LRR‐RK and LRR‐RP signaling pathways.

### nlp20‐induced elevated ROS production in *bik1* genotypes is only partially SA dependent

It has been reported that SA positively regulates ROS production in response to flg22 (Yi *et al*., 2014). Therefore, elevated SA levels in the *bik1* mutant (Veronese *et al*., 2006; Fig. 5c) could potentially explain the higher response to nlp20 in this genotype. To test this, we obtained *bik1 sid2* seeds (Laluk *et al*., 2011). *Sid2* (*SALICYLIC ACID INDUCTION‐DEFICIENT 2*) is deficient in SA biosynthesis. Overall, *sid2* and *sid2 bik1* plants showed reduced ROS responses to both nlp20 and flg22 compared with the *bik1* mutant (Fig. S6). Moreover, the response to nlp20 in *bik1 sid2* was still elevated relative to the response in *sid2* (Fig. S6). However, we did not observe statistical significant differences between the *bik1* and the *sid2 bik1* or the *sid2* and the *sid2 bik1* mutants. Therefore, it appears that SA production does, if at all, not fully account for the elevated ROS response to nlp20 in the *bik1* mutant. Similarly, elevated SA levels in *bik1* mutants may not fully explain the differential behaviour of these plants to RK and RP ligands.

### BIK1 phosphorylation differs after flg22 and nlp20 treatment

**Figure 7:**
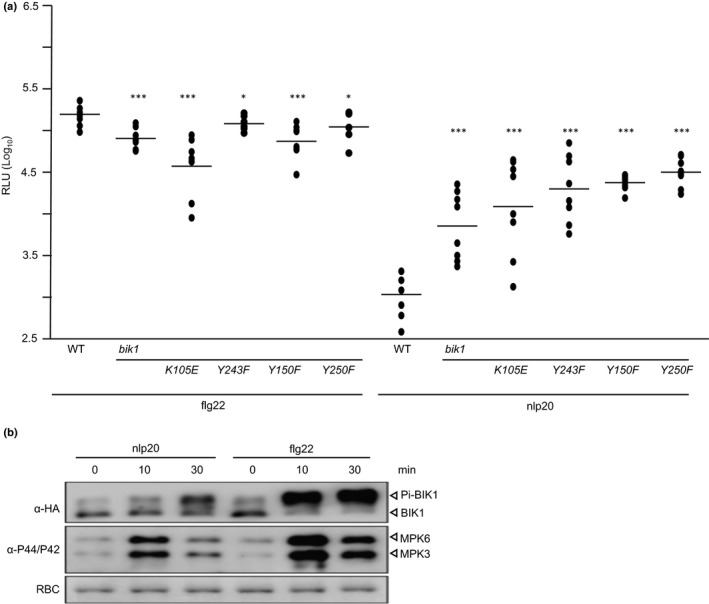
BIK1 kinase activity and phosphorylation are required for both nlp20‐ and flg22‐induced ROS production. (a) Reactive oxygen species (ROS) production (relative fluorescence units, RLU) was determined in leaf discs of Arabidopsis wild‐type (WT) plants or the mutant lines *bik1*,* bik1/BIK1_K105E*,* bik1/BIK1_Y243F*,* bik1/BIK1_Y150F* and *bik1/BIK1_Y250F* each treated with 0.5 μM flg22 or nlp20, respectively. Peak value minus background value are shown as dots (*n *≥* *6) and means are presented as lines. Asterisks indicate significant differences to the wild‐type control treatment as determined by Student's *t*‐test: *, *P *<* *0.05; ***, *P *<* *0.001. The experiments were performed three times with similar results. (b) Arabidopsis protoplasts were transformed transiently with a BIK1‐HA construct and subsequently treated with 1 μM nlp20 or 100 nM flg22 for the times indicated. Isolated protein extracts were subjected to western blot analysis using either hemagglutinin (HA) antisera to detect nonphosphorylated or phosphorylated BIK1 (Pi‐BIK1) or the phospho‐p44/42 MAP kinase antibody to detect activated mitogen‐activated protein kinases (MAPKs). Protein loading control was shown by Coomassie Brilliant Blue staining for RuBisCO (RBC) (bottom panel).

BIK1 exhibits hallmarks of a classical serine/threonine protein kinase, has auto‐phosphorylation activity, and its kinase activity is required for positive regulation of flg22‐induced immune activation (Lu *et al*., 2010; Zhang *et al*., 2010). A mutation in the ATP binding pocket (K105E) as well as a tyrosine phosphorylation site mutant (Y150F) abolishes BIK1 catalytic activity and flg22 signaling (Zhang *et al*., 2010; Lin *et al*., 2014), indicating that BIK1 kinase activity is required for BIK1 function in flg22‐induced defenses (Fig. 7a). We have challenged *bik1* plants, expressing these catalytically inactive BIK1 mutants with nlp20 and observed similar levels of ROS production as in *bik1* mutants. Therefore, loss of BIK1 kinase activity phenocopies *bik1* mutant phenotypes, suggesting that BIK1 catalytic activity is required for the negative regulatory activity of BIK1 in LRR‐RP‐mediated immune signaling (Figs 7a, S5).

Flg22 treatment results in BAK1‐dependent phosphorylation of BIK1 at tyrosine residues Y243 and Y250, and these phosphorylation sites are important for BIK1 function in flg22 signaling and bacterial resistance (Lin *et al*., 2014). We observed that *bik1* mutants producing BIK1 Y243F and BIK1 Y250F mutant proteins mounted higher ROS levels upon nlp20‐treatment than wild‐type plants (Figs 7a, S5c), suggesting that these two phosphosites are important for both flg22‐ and nlp20‐induced immune signaling.

Flg22‐ and elf18‐mediated BIK1 phosphorylation can be detected as a shift in BIK1 molecular size (Lu *et al*., 2010; Zhang *et al*., 2010; Kadota *et al*., 2014), which is absent in *bik1* mutants complemented with BIK1 Y150F and BIK1 Y250F (Lin *et al*., 2014). We found that nlp20 application also causes a BIK1 protein mobility shift (Fig. 7b). However, onset of BIK1 phosphorylation was substantially delayed when compared with flg22 treatment. Similarly, amplitude of BIK1 phosphorylation was lower upon nlp20 treatment (Fig. 7b).

Altogether, our data suggest that BIK1 kinase activity is important for both flg22‐ and nlp20‐induced immune signaling, but timing and extent of BIK1 phosphorylation differ substantially upon treatment with either pattern.

## Discussion

It has been a longstanding assumption that different microbial patterns, albeit recognized by different classes of PRRs, trigger ‘generic’ intracellular signaling pathways to stimulate immunity to invading pathogens. Hence, MAMPs are believed to feed into converging signaling networks that may differ in certain components, but nevertheless give rise to the same physiological output, broadly termed plant immunity. Immunogenic patterns can be structurally quite diverse and include peptides (e.g. flg22, elf18), carbohydrates (e.g. peptidoglycans, chitin), lipids (e.g. lipopolysaccharides) and other molecules (e.g. ATP) (Gust *et al*., 2012, 2017; Albert, 2013; Ranf, 2017). Most of these molecules do indeed trigger a conserved set of immunity‐associated early and late responses, including the accumulation of phytohormones and ROS, the activation of MAPK cascades or reprogramming of gene expression and defense metabolite production (Bigeard *et al*., 2015; Saijo *et al*., 2018). However, previous studies have already highlighted differences in the activation of plant defenses due to different stimuli. For instance, Arabidopsis seedling growth retardation was observed after flg22 or elf18 treatment (Gomez‐Gomez & Boller, 2000; Zipfel *et al*., 2006), but not after nlp20‐treatment (Böhm *et al*., 2014b). It has also been shown that calcium signatures differ according to the immunogenic stimulus applied (Blume *et al*., 2000; Keinath *et al*., 2010). In our systematic comparative analysis of flg22‐ and nlp20‐induced immune responses, we have also observed spatial‐temporal differences in cytoplasmic calcium accumulation with nlp20 signals being delayed compared with flg22‐triggered Ca^2+^ bursts and limited to the meristematic zone in the root. Whether this nlp20‐specific calcium signature translates into delayed kinetics of activation of downstream responses is presently unclear. It has become clear, however, that such differences in timing and extent of cellular responses apparently do not affect immunity to microbial infection in general. Both, LRR‐RP and LRR‐RK‐mediated immune signaling pathways eventually result in PTI activation (Zipfel *et al*., 2004; Albert *et al*., 2015), suggesting a substantial degree of molecular plasticity underlying immune activation through structurally related, yet different LRR‐type pattern recognition receptor systems.

A striking observation was that nlp20 was an effective trigger of camalexin accumulation but flg22 was not. Flg22 treatment induces miR393, which blocks accumulation of transcripts encoding camalexin biosynthetic enzymes and, subsequently, camalexin production (Robert‐Seilaniantz *et al*., 2011). It has been suggested that suppression of camalexin biosynthesis may re‐direct secondary metabolite synthesis towards glucosinolates, which are considered the most effective anti‐microbial compounds for plant resistance against biotrophic pathogens. However, flg22 treatment was demonstrated to result in rather decreased glucosinolate production (Clay *et al*., 2009). We found that levels of the most abundant indole glucosinolate indol‐3‐ylmethyl glucosinolate (I3M) were not significantly altered upon neither flg22 nor nlp20 stimulation, and transcript levels of the glucosinolate marker gene *CYP81F2* did not differ between the two treatments (Fig. S7).

A recent model considers LRR‐RP/SOBIR1 heterodimers as bi‐partite LRR‐RKs that are mechanistically equivalent to the latter (Gust & Felix, 2014). This view is compelling given that both receptor types: require stimulus‐dependent recruitment of the co‐receptor BAK1; mediate activation of generic immunity‐associated defenses; and confer immunity to microbial infection. However, our in‐depth assessments of signaling outputs mediated through activation of related LRR‐type PRRs, RLP23 and FLS2 (and also RLP42 and EFR), have challenged this hypothesis as we have found both quantitative and qualitative differences in signaling pathways and defense responses mediated through either receptor system. If such observed differences are representative of whole classes of PRRs or reflect an even deeper diversification of signaling webs that is rather characteristic for any individual receptor remains to be shown. Of note, LRR‐RKs FLS2 and EFR mediate activation of largely overlapping gene expression patterns and immunity‐associated responses (Zipfel *et al*., 2006).

Our study further reveals that suppression of LRR‐type PRR activity in the absence of ligand is mechanistically very similar and occurs largely at the level of the co‐receptor BAK1. BIR2 and PP2A, known negative regulators of BAK1 (Halter *et al*., 2014; Segonzac *et al*., 2014), suppress flg22‐ and nlp20‐triggered immune activation in untreated plants. BAK1 is widely accepted to act as a co‐receptor in LRR‐RK‐ as well as in LRR‐RP‐type PRR complexes (Böhm *et al*., 2014b; Gust & Felix, 2014; Boutrot & Zipfel, 2017; Saijo *et al*., 2018). In both cases BAK1 is recruited to the receptor complex after ligand binding (Albert *et al*., 2015; Postma *et al*., 2016). Importantly, the relevance of BAK1 phosphorylation sites is conserved between flg22‐ and nlp20‐pathways, suggesting a large degree of conservation of very early signaling events occurring after ligand perception independent of the receptor type involved. Nevertheless, FLS2 (largely dependent on SERK3/BAK1) (Chinchilla *et al*., 2007; Heese *et al*., 2007; Roux *et al*., 2011) and RLP23 (dependent on SERK1, SERK3/BAK1, SERK4/BKK1) (Albert *et al*., 2015) exhibit different requirements for members of the SERK protein family, a phenomenon that is commonly found for this family of co‐receptors (Ma *et al*., 2016).

CPK28 and heterotrimeric G‐proteins have a role in turnover and stabilization of the receptor‐like cytoplasmic kinase BIK1, respectively (Monaghan *et al*., 2014; Liang *et al*., 2016; Wang *et al*., 2018). Intriguingly, whereas these proteins regulate both LRR‐RK (FLS2, EFR) and LRR‐RP signaling (RLP23, RLP42) in a similar fashion (Figs 4c,d, 6), BIK1 itself has opposing roles in the two pathways. *bik1* mutants showed an enhanced oxidative burst after treatment with the LRR‐RP ligands nlp20 and PG3; this is in contrast with its positive regulatory roles in flg22 and elf18 signaling (Lu *et al*., 2010; Zhang *et al*., 2010), suggesting a different mode of action of BIK1 in the respective signaling networks. BIK1 not only negatively regulates nlp20/RLP23‐mediated immune activation, but also aphid resistance (Lei *et al*., 2014) and hormone Brassinolide (BL)‐induced plant growth (Lin *et al*., 2013). The inverse modulation of flg22 and BL signaling pathways is mediated through phosphorylation of BIK1 by BRI1 in a BAK1‐independent manner (Lin *et al*., 2013). By contrast, flg22‐induced phosphorylation of BIK1 occurs in a BAK1‐dependent manner (Lu *et al*., 2010; Lin *et al*., 2013). Our experiments revealed that BIK1 also undergoes rapid phosphorylation upon treatment with nlp20, but timing and strength differed compared with flg22‐treatment. It is conceivable that differences in nlp20 and flg22‐induced BIK1 phosphorylation patterns may be causal for the differential activities of BIK1 observed in these two immune signaling pathways. Some molecular scenarios that may account for the observed differential regulatory roles of BIK1 in RK and RP‐mediated immune signaling are addressed below.

As BIK1 is a direct phosphorylation target of CPK28 (Monaghan *et al*., 2014), which itself is negatively regulating both flg22 and nlp20 signaling, future work should address whether BIK1 undergoes stimulus‐dependent differential phosphorylation that is either indeed mediated by CPK28 or possibly other related kinases. Similarly, nlp20‐triggered CPK28‐dependent differential phosphorylation of the two homologous E3 ligases PUB25 (PLANT U‐BOX PROTEIN 25) and PUB26 that mark BIK1 for degradation via the proteasome after flg22 treatment (Wang *et al*., 2018), should be considered. For nlp20‐treatment, differential phosphorylation of either BIK1 directly or PUB25/26 may result in signaling events that do not trigger BIK1 decay. Alternatively, it cannot be ruled out that CPK28 also has additional phosphorylation targets that impact BIK1 function during RP‐mediated immune responses.

Whether BIK1 physically associates with SOBIR1 in a ligand (in)dependent fashion and whether there is ligand‐induced transphosphorylation of these two kinase‐active proteins also remains to be shown. In this respect it will also be interesting to investigate whether BIK1 phosphorylates substrates (such as RBOHD) in a nlp20‐specific manner, as previously shown upon flg22‐treatment (Kadota *et al*., 2014; Li *et al*., 2014). Similarly, yet unidentified BIK1‐interacting proteins (substrates) may explain the differential involvement of BIK1 in flg22/FLS2 and nlp20/RLP23‐mediated immune signaling.

Lal *et al*. (2018) recently reported that, in addition to its known plasma membrane localization, BIK1 also localizes to the nucleus. BIK1 interacts with and phosphorylates transcription factors that are known to be involved in SA and jasmonic acid signaling (WRKY33, 50, and 57) *in vitro*. It will be of interest to determine the importance of the nuclear activity of BIK1 in immune signaling *in vivo* and if this is similar during RP and RK signaling. Differential activation of transcription factors by BIK1 is another possible explanation for the differential role of BIK1 in RP and RK signaling.

Taken together, our studies have revealed the rather unexpected insight that LRR‐RK‐ and LRR‐RP‐mediated signaling networks differ partially in architecture and output while bringing about basal resistance (PTI) to microbial infection. We would like to note, however, that while we find LRR‐type‐specific response patterns, we would not rule out the existence of individual differences in immune signaling and output even between PRRs of the same structural type. This is, for example, exemplified by our findings that PG3 triggers only a small ROS burst in Arabidopsis (Figs 6a, S4), whereas LRR‐RP ligands nlp20 or SCFE1 do so in a significant manner. What is equally important, is the insight that basal immunity against microbial infection (triggered by all patterns tested) can be brought about in different ways. In other words, immune signaling networks display a rather high degree of plasticity in how immune activation can be achieved as reviewed recently (Wu *et al*., 2018). A second conclusion from our studies is that RP/SOBIR1 heteromers should not merely be considered bipartite RKs as proposed previously (Gust & Felix, 2014). Lastly, we have ascribed an unexpected negative regulatory role of the cytoplasmic protein kinase BIK1, which is known as a positive regulator of flg22‐induced ROS burst, in LRR‐RP‐mediated immune activation. In summary, we conclude that LRR‐RK‐ and LRR‐RP‐type PRRs both mediate immunity to microbial infections, but make receptor type‐specific use of the signaling capabilities that plants have evolved to cope with microbial infection.

## Author contributions

TN and AAG conceived and designed the study and analyzed the data. W‐LW, LZ, RP, MZ, RB, XM, EK, GG, JK, MS, JALvK, RH performed the experiments and analyzed the data. LS, AP and CZ provided unpublished material and analyzed the data. TN, AAG, DW, W‐LW, RP and LZ wrote the article. All the authors critically read and commented on the article and approved of its final version for submission.

## Supporting information

Please note: Wiley Blackwell are not responsible for the content or functionality of any Supporting Information supplied by the authors. Any queries (other than missing material) should be directed to the *New Phytologist* Central Office.

**Fig. S1** Time course of flg22 and nlp20‐triggered membrane depolarization.
**Fig. S2** Spatiotemporal analysis of calcium responses to nlp20 and flg22.
**Fig. S3** Time course of flg22 and nlp20‐triggered ROS production and MAPK activation.
**Fig. S4** Immune responses triggered by RK‐ligands flg22 and elf18 compared with RP‐ligands nlp20 and PG3.
**Fig. S5** Time course of flg22 and nlp20‐triggered ROS production in *bak1* and *bik1* mutant lines.
**Fig. S6** Flg22 and nlp20‐triggered ROS production in *bik1* and *sid2* mutant lines.
**Fig. S7** Indole glycosinolate levels remain unchanged upon flg22 and nlp20 treatment.
**Methods S1** Supplemental methods.
**Table S1** *Arabidopsis thaliana* mutant and transgenic lines used in this study.
**Table S3** Examples of genes specifically upregulated by flg22 or nlp20 categorized by GO terms.

**Table S2** GO term list of RNA‐seq data obtained from *Arabidopsis thaliana* treated with flg22 or nlp20.

**Video S1** Time‐lapse recording of cytoplasmic Ca^2+^ elevations in an R‐GECO1‐expressing root treated with flg22.

**Video S2** Time‐lapse recording of cytoplasmic Ca^2+^ elevations in an R‐GECO1‐expressing root treated with nlp20.
