# Supplementary material for: Comparing Arabidopsis receptor kinase and receptor protein‐mediated immune signaling reveals BIK1‐dependent differences
Source: New Phytol. 2018 Oct 25;221(4):2080–95. doi: 10.1111/nph.15497 (PMC6367016; doi:10.1111/nph.15497)
Supplement: Supplementary file 1 — Fig. S1 Time course of flg22 and nlp20‐triggered membrane depolarization. Fig. S2 Spatiotemporal analysis of calcium responses to nlp20 and flg22. Fig. S3 Time course of flg22 and nlp20‐triggered ROS production and MAPK activation. Fig. S4 Immune responses triggered by RK‐ligands flg22 and elf18 compared with RP‐ligands nlp20 and PG3. Fig. S5 Time course of flg22 and nlp20‐triggered ROS production in bak1 and bik1 mutant lines. Fig. S6 Flg22 and nlp20‐triggered ROS production in bik1 and sid2 mutant lines. Fig. S7 Indole glycosinolate levels remain unchanged upon flg22 and nlp20 treatment. Methods S1 Supplemental methods. Table S1 Arabidopsis thaliana mutant and transgenic lines used in this study. Table S3 Examples of genes specifically upregulated by flg22 or nlp20 categorized by GO terms. [file NPH-221-2080-s001.pdf]

***New Phytologist* Supporting Information**

Article title: Comparing Arabidopsis receptor kinase and receptor protein-mediated immune signaling reveals BIK1-dependent differences

Authors: Wei-Lin Wan, Lisha Zhang, Rory Pruitt, Maricris Zaidem, Rik Brugman, Xiyu Ma, Elzbieta Krol, Artemis Perraki, Joachim Kilian, Guido Grossmann, Mark Stahl, Libo Shan, Cyril Zipfel, Jan A.L. van Kan, Rainer Hedrich, Detlef Weigel, Andrea A. Gust, Thorsten Nürnberger

Article acceptance date: 11 September 2018

The following Supporting Information is available for this article:

Fig. S1 Time course of flg22 and nlp20-triggered membrane depolarization.

Fig. S2 Spatiotemporal analysis of calcium responses to nlp20 and flg22.

Fig. S3 Time course of flg22 and nlp20-triggered ROS production and MAPK activation.

Fig. S4 Immune responses triggered by RK-ligands flg22 and elf18 compared to RP-ligands nlp20 and PG3.

Fig. S5 Time course of flg22 and nlp20-triggered ROS production in *bak1* and *bik1* mutant lines.

Fig. S6 Flg22 and nlp20-triggered ROS production in *bik1* and *sid2* mutant lines.

Fig. S7 Indole glycosinolate levels remain unchanged upon flg22 and nlp20 treatment.

Table S1 *Arabidopsis thaliana* mutant and transgenic lines used in this study.

Table S2 GO term list of RNA-seq data done in *Arabidopsis thaliana* under elicitor treatment.

Table S3 Examples of genes specifically upregulated by flg22 or nlp20 categorized by GO terms.

Video/Movie S1 Time-lapse recording of cytoplasmic Ca^{2+} elevations in an R-GECO1-expressing root treated with flg22.

Video/Movie S2 Time-lapse recording of cytoplasmic Ca^{2+} elevations in an R-GECO1-expressing root treated with nlp20.

Methods S1 Supplemental Methods

Fig. S1 Time course of flg22 and nlp20-triggered membrane depolarization.
Leaves of Arabidopsis Col-0 wild type plants were treated with 100 nM nlp20 or 10 nM flg22, and changes in membrane depolarization (ΔV) were monitored continuously. The response to flg22 was generally higher than nlp20, but the difference was not statistically significant.

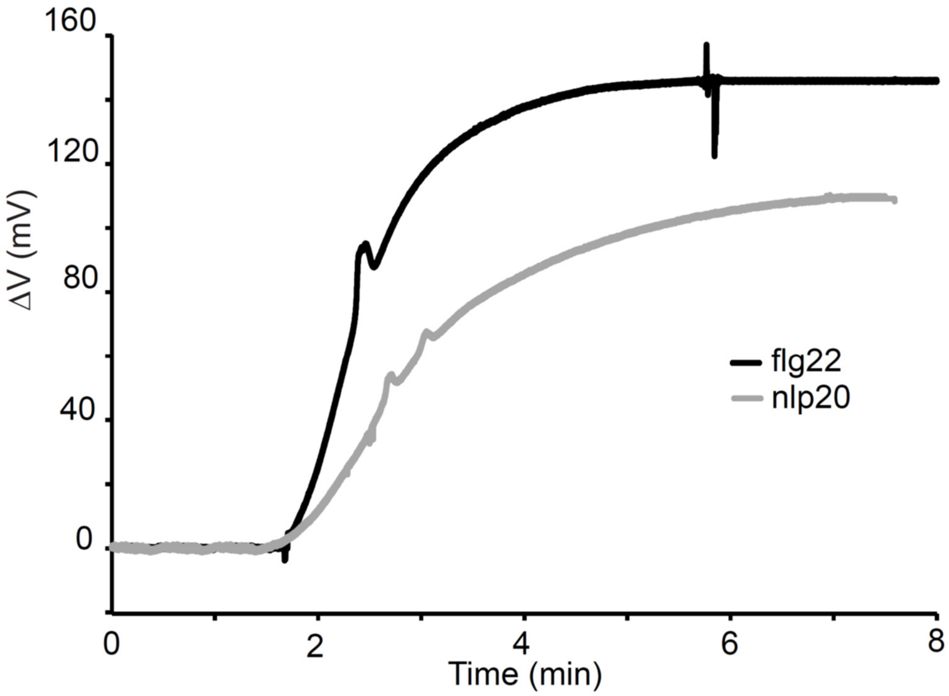

Fig. S2 Spatiotemporal analysis of calcium responses to nlp20 and flg22. Kymograph analysis of R-GECO1 signal intensities upon root treatment with 10 µM nlp20 **(a)** or 10 µM flg22 **(b)**. Signal intensities are normalized for the average signal intensity of the baseline (25 frames). The cartoon above illustrates the different developmental zones of the root along the spatial (horizontal) axes of the kymographs. M, meristem; TZ, transition zone; EZ, elongation zone; DZ, differentiation zone. The calibration bar on the right indicates signal intensities with blue color indicating low and red color indicating high intensity.

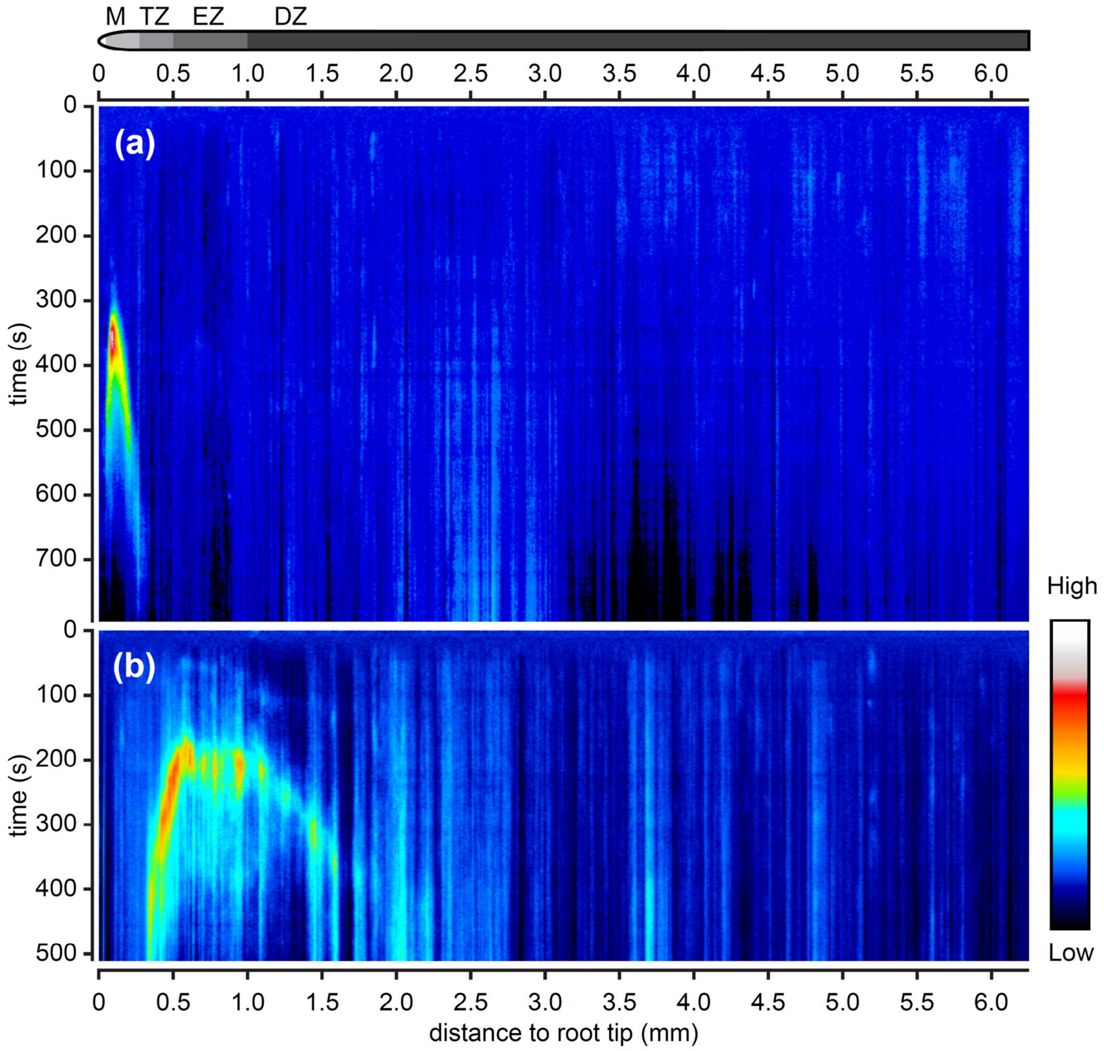

Fig. S3 Time course of flg22 and nlp20-triggered ROS production and MAPK activation.

Arabidopsis leaf discs were treated with flg22 or nlp20 (100 nM or 1 µM), or water as control (mock), and ROS production **(a)** or MAPK activation **(b)** was monitored over time as described in Fig. 1. Bars in **(a)** present means ± SD (n≥6) of relative fluorescence units (RLU). **(c)** Arabidopsis wild-type seedlings were treated with water or 0.5 µM nlp20 or flg22 for 1 and 6 hours, and isolated RNA was subjected to RNA sequencing as described in Fig. 2. Given are the fold changes (Log2) of *MLO12* transcript levels compared the water control. For qRT-PCR, leaves of Arabidopsis wild type plants were infiltrated with water (mock), 0.5 µM flg22 or 5 µM nlp20, and collected 6 hours after treatment. Relative expression of the *MLO12* gene was normalized to the levels of *EF-1α* transcript and calibrated to the levels of mock treatment.

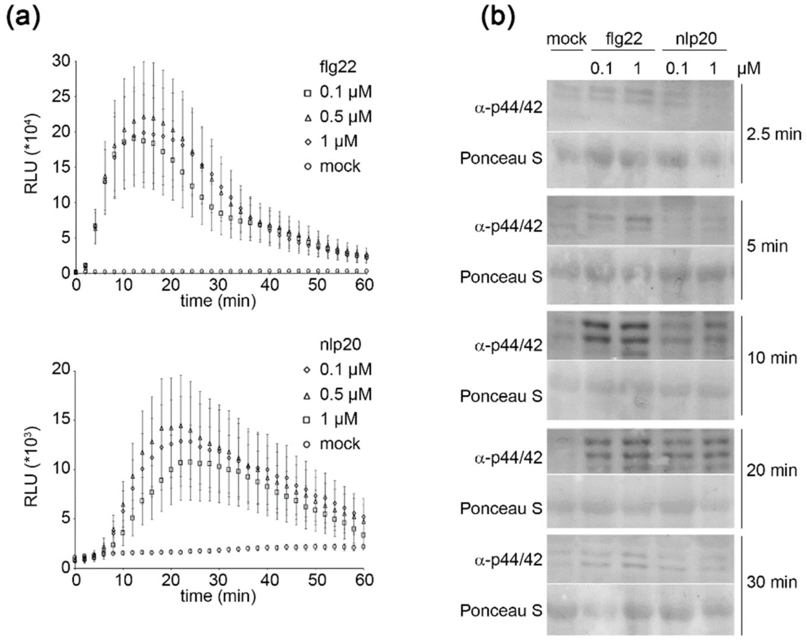

(c)

RNAseq

treatment / time	flg22 0.5 µM	nlp20 0.5 µM
1h	3,5	0
6h	3,8	0

RT-qPCR

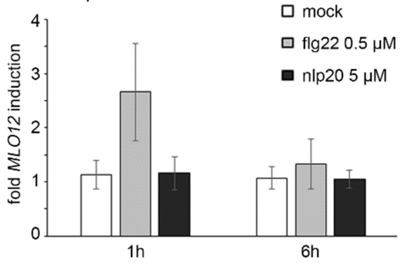

Fig. S4 Immune responses triggered by RK-ligands flg22 and elf18 compared to RP-ligands nlp20 and PG3.

(a) Arabidopsis leaf discs were treated with 100 nM flg22, elf18, nlp20, or PG3, or water as control (mock), and ROS production was monitored over time. Bars present means ± SD (n=6) of relative luminescence units (RLU). **(b)** Arabidopsis leaf discs were treated with flg22, elf18, nlp20, or PG3, or water as control (mock), and ethylene production was measured at 3 and 6 hours post incubation. Bars present means ± SD (n=3). For each time point, different letters mean significant differences (P<0.05) by Student's *t*-test. **(c)** *PR1* expression by qRT-PCR. Arabidopsis leaves were infiltrated with water (mock) or 500 nM flg22, elf18, nlp20, or PG3 and harvested 6 hours post infiltration. Transcript levels of *PR1* were normalized to the levels of *EF-1α* and calibrated to the levels of mock treatment. Bars present means ± SD (n=3). Different letters mean significant differences (P<0.05) by Student's *t*-test. **(d)** Callose production in Arabidopsis leaves infiltrated with water (mock), each 1 μM flg22, elf18, nlp20, or PG3. Callose deposition was visualized with aniline blue 16 hours after infiltration and evaluated with fluorescence microscopy (left panel). Scale bars, 500 μm. The diagram (right panel) depicts the amount of callose deposits and the bars present means ± SD from 5 pictures. Different letters mean significant differences (P<0.01) by Student's *t*-test. **(e)** *CYP71A13A* and *PAD3* expression by qRT-PCR. Arabidopsis leaves were infiltrated with water (mock), each 0.5 μM flg22, elf18, nlp20, or PG3 and harvested 6 hours post infiltration. Transcript levels were normalized to the levels of *EF-1α* and calibrated to the levels of mock treatment. Bars present means ± SD (n=3). Different letters mean significant differences (P<0.05) by Student's *t*-test. These data were extracted from Fig. 6b and are shown as separate panel here.

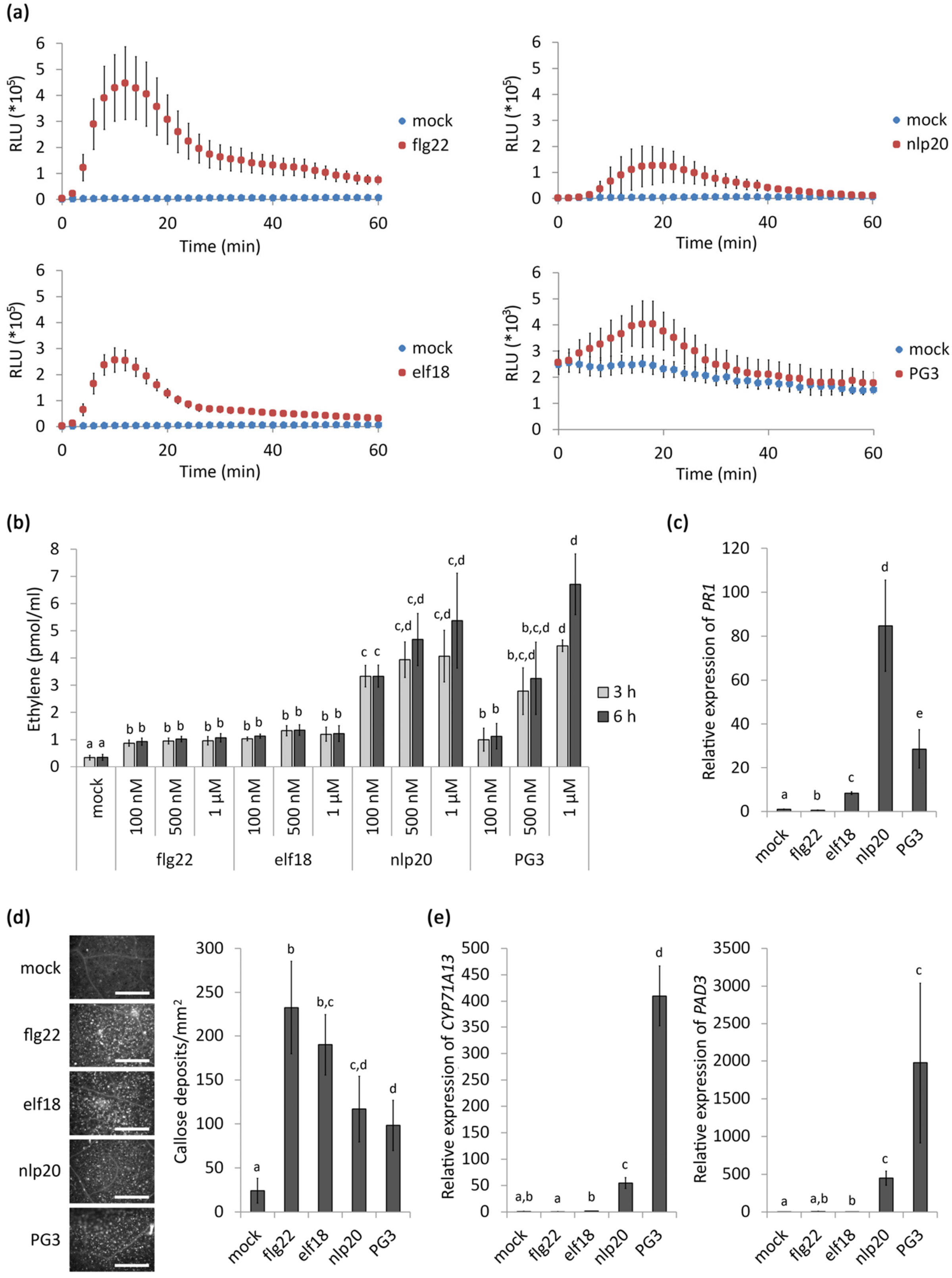
(a)
RLU (*10^5)
Time (min)
mock
flg22
nlp20
elf18
RLU (*10^3)
PG3
(b)
Ethylene (pmol/ml)
3 h
6 h
mock
100 nM
500 nM
1 µM
flg22
elf18
nlp20
PG3
(c)
Relative expression of PR1
(d)
Callose deposits/mm²
(e)
Relative expression of CYP71A13
Relative expression of PAD3

Fig. S5 Time course of flg22 and nlp20-triggered ROS production in *bak1* and *bik1* mutant lines. Arabidopsis leaf discs of the indicated *bak1* **(a, b)** or *bik1* **(b, c)** mutant lines were treated with water (mock), 500 nM flg22 **(a, c)** or 500 nM nlp20 **(b, d)** and ROS production was monitored over time as described in Fig. 1. Data present means $\pm$ SD (n$\geqslant$6) of relative fluorescence units (RLU).

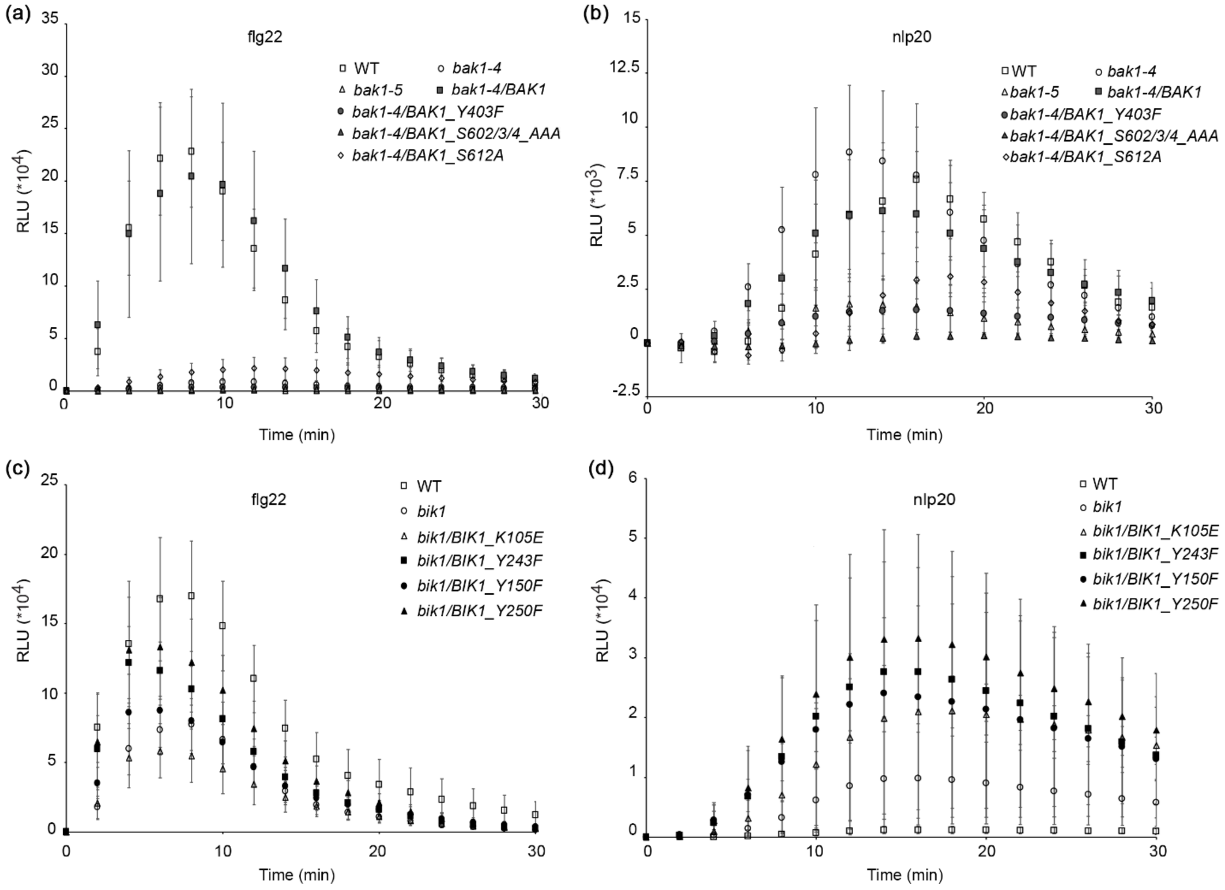

Fig. S6 Flg22 and nlp20-triggered ROS production in *bik1* and *sid2* mutant lines.

Leaf pieces of wild-type, *bik1, sid2,* or *bik1 sid2* plants were treated with water, 500 nM flg22, or 500 nM nlp20, and ROS accumulation was determined as in Fig. 1. The composite 15 independent experiments with each point representing the mean (n=8) total ROS production over 1 h. The mean total ROS production (relative fluorescence units, RLU) for the set of experiments is indicated by a horizontal line. Within each treatment, different letters indicate significant differences (*P*<0.05) using Student's *t*-test for all possible individual comparisons.

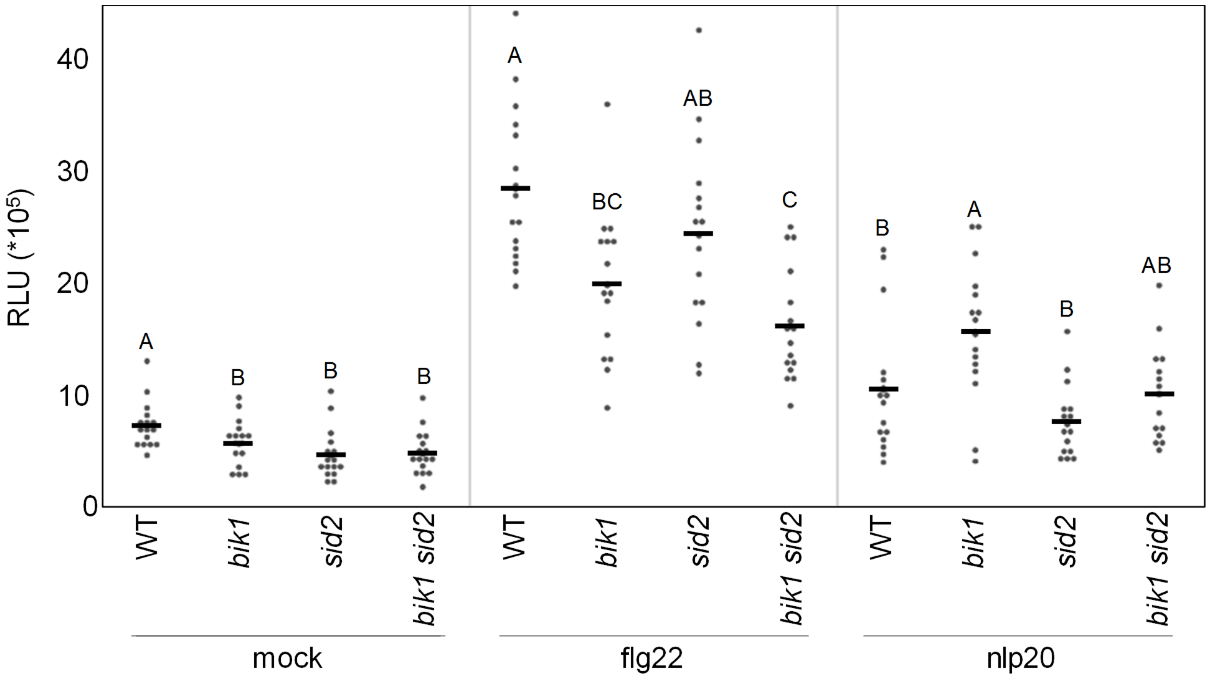

Fig. S7 Indole glycosinolate levels remain unchanged upon flg22 and nlp20 treatment.

(a) Levels of the indole glycosinolate indol-3-ylmethyl glucosinolate (I3M) were determined in leaves infiltrated with 1 µM flg22 or nlp20 (also 0.1 µM for 48 hours), or water (mock) and harvested after 12 and 48 hours. Bars (nmol I3M/g fresh weight) present average values ± SD (n = 2). **(b)** Arabidopsis wild-type seedlings were treated with water or 0.5 µM nlp20 or flg22 for 1 and 6 hours, and isolated RNA was subjected to RNA sequencing. Given are the fold changes (log_2) of *CYP81F2* transcript levels compared the water control.

(a)

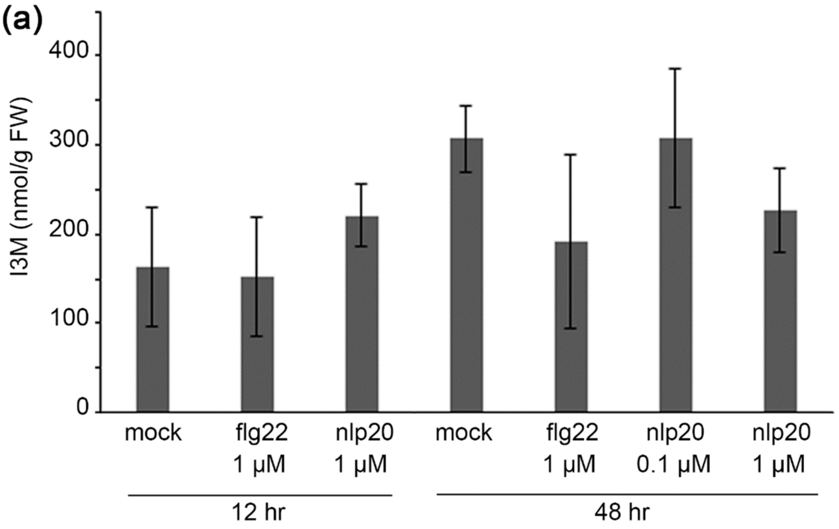

(b) *CYP81F2* transcript levels

time \ treatment	flg22	nlp20
1h	7,78	6,99
6h	5,58	5,59

Table S1 *Arabidopsis thaliana* mutant and transgenic lines used in this study.

**Line Name**	**Locus**	**Description**	**Ref.**
rlp23-1 (SALK_034225)	At2g32680	insertion	Albert *et al.* (2015)
sobir1-12 (SALK_050715)	At2g31880	insertion	Gao *et al.* (2009)
bak1-4 (SALK_116202)	At4g33430	insertion	Heese *et al.* (2007)
bak1-5	At4g33430	substitution (C408Y)	Schwessinger *et al.* (2011)
bak1-4/BAK1		*pBAK1:BAK* in *bak1-4*	Heese *et al.* (2007)
bak1-4/ BAK1_Y403F		*pBAK1:BAK_Y403F* in *bak1-4*	Perraki *et al.* (2018)
bak1-4/ BAK1_S602/3/4AAA		*pBAK1:BAKS602/3/4AAA* in *bak1-4*	Perraki *et al.* (2018)
bak1-4/BAK1_S612A		*pBAK1:BAK_S612A* in *bak1-4*	Perraki *et al.* (2018)
bir2-1 (GK-793F12)	At3g28450	insertion	Halter *et al.* (2014)
pp2a-a1 (SALK_059903)	At1g25490	insertion	Segonzac *et al.* (2014)
pp2a-c4 (SALK_035009)	At3g58500	insertion	Segonzac *et al.* (2014)
cpk28-1 (GK_523B08)	At5g66210	insertion	Monaghan *et al.* (2014)
bik1 (SALK_005291)	At2g39660	insertion	Lu *et al.* (2010)
pbl1 (SAIL_1236_D07)	At3g55450	insertion	Zhang *et al.* (2010)
bik1 pbl1		double mutant	Zhang *et al.* (2010)
xlg2-1 (SALK_062645)	At4g34390	insertion	Ding *et al.* (2008); Liang *et al.* (2016)
agb1-2 (SALK_061896)	At4g34460	insertion	Ullah *et al.* (2003); Liang *et al.* (2016)
agg1 agg2	At3g63420/ At3g22942	backcrossing *agg1-1w* with Col-0 to obtain *agg1-1c*, crossing *agg1-1c* with *agg2-1* to obtain double mutant	Trusov *et al.* (2007); Liang *et al.* (2016)
bik1/BIK_K105E		*pBIK1:BIK1_K105E:HA* in *bik1*	Lin *et al.* (2014)
bik1/BIK1_Y243F		*pBIK1:BIK1_Y243F:HA* in *bik1*	Lin *et al.* (2014)
bik1/BIK1_Y150F		*pBIK1:BIK1_Y150F:HA* in *bik1*	Lin *et al.* (2014)
bik1/BIK1_Y250F		*pBIK1:BIK1_Y250F:HA* in *bik1*	Lin *et al.* (2014)
bik1 sid2	At2g39660/ At1g74710	double mutant	Laluk *et al.* (2011)

Table S2 GO term list of RNA-seq data from *Arabidopsis thaliana* treated with flg22 or nlp20. Singular Enrichment Analysis (SEA) was perform by agriGO for transcripts differentially expressed after 0.5 μM flg22 or 0.5 μM nlp20 treatment for 1 h **(a-f)** and 6 h **(g-k)**, GO terms with FDR ⩽ 0.5 are shown. Analysis of 1,638 transcripts up-regulated only by flg22 **(a)**. Analysis of 22 transcripts up-regulated only by nlp20 **(b)**. Analysis of 1,492 transcripts up-regulated by both flg22 and nlp20 **(c)**. Analysis of 1,892 transcripts down-regulated only by flg22 **(d)**. Analysis of 20 transcripts down-regulated only by nlp20 **(e)**. Analysis of 139 transcripts down-regulated by both flg22 and nlp20 **(f)**. Analysis of 1,699 transcripts up-regulated only by flg22 **(g)**. Analysis of 116 transcripts up-regulated only by nlp20 **(h)**. Analysis of 1,184 transcripts up-regulated by both flg22 and nlp20 **(i)**. Analysis of 2,651 transcripts down-regulated only by flg22 **(j)**. Analysis of 17 transcripts down-regulated only by nlp20 **(k)**. Analysis of 140 transcripts down-regulated by both flg22 and nlp20 **(l)**. P: Biological process. F: Molecular function. C: Cellular component. BG/Ref: Background/Reference. FDR: False Discover Rate. Relation and significant levels of GO terms are shown as figures with significant levels and arrow types diagram.

file uploaded as separate .xls file

Table S3 Examples of genes specifically upregulated by flg22 or nlp20 categorized by GO terms.

**1-hr specifically up-regulated by flg22**	**6-hr specifically up-regulated by flg22**	**6-hr specifically up-regulated by nlp20**
**defense response**	**defense response**	**response to ethylene**
AT3G53260 ATPAL2	AT3G52430 PAD4	AT2G05520 ATGRP-3
AT3G45290 ATMLO3	AT1G64280 NPR1	AT5G19880
AT2G17480 ATMLO8	AT3G48090 EDS1	AT5G40990 GLIP1
AT5G56030 HSP81.2	AT5G04720 ADR1-L2	AT3G23230 ATERF98
**immune response**	**immune response**	**response to reactive oxygen species**
AT4G12010 DSC1	AT5G51700 PBS2	AT4G26070 ATMEK1
AT5G46520 ACQOS	AT4G26090 RPS2	AT3G12580 ATHSP70
AT5G64900 ATPEP1	AT3G07040 RPM1	AT2G26150 ATHSFA2
AT1G66340 ETR1	AT1G63750 (TIR-NBS-LRR)	
**regulation of signal transduction**	**regulation of signal transduction**	
AT4G18950 BHP	AT1G35670 ATCDPK2	
AT5G60410 ATSIZ1	AT3G15210 ERF4	
AT2G22430 ATHB6	AT3G02140 TMAC2	
AT3G04580 EIN4	AT3G45640 MPK3	
**protein modification process**	**cellular protein catabolic process**	
AT4G05320 UBQ10	AT5G58290 RPT3	
AT5G07460 ATMSRA2	AT4G05050 UBQ11	
AT1G10560 PUB18	AT4G38630 RPN10	
AT2G39550 PGGT-I	AT4G31300 PBA1	
	**protein modification process**	
	AT5G24240 MOP9.5	
	AT4G05050 UBQ11	
	AT4G35310 CPK5	
	AT3G62260 PP2C	

Video/Movie S1 Time-lapse recording of cytoplasmic Ca^{2+} elevations in an R-GECO1-expressing root treated with flg22.

Video/Movie S2 Time-lapse recording of cytoplasmic Ca^{2+} elevations in an R-GECO1-expressing root treated with nlp20.

Methods S1 Supplemental Methods

Plant Material

Arabidopsis plants were grown on soil or half-strength Murashige and Skoog (MS) medium as described (Brock *et al.*, 2010). Plants were grown in climate chambers under short-day conditions (8 h : 16 h, light : darkness, 150 µmol/cm^{2}s white fluorescent light, 40-60 % humidity, 22 °C). All mutants used in this study are in *Arabidopsis thaliana* accession Col-0 background (listed in Table S1).

Elicitors

Flg22, nlp20 and elf18 peptides were synthesized according to the published sequences (Felix *et al.*, 1999; Kunze *et al.*, 2004; Böhm *et al.*, 2014b) by Genscript Inc., prepared as 10 mM stock solutions in ddH_2O, and diluted in ddH_2O prior to use. *Botrytis cinerea* PG3 was purified from culture filtrates of *Pichia pastoris* as described previously (Kars *et al.*, 2005).

Ion Flux Measurements

Membrane potential recordings were performed in 5-7-week-old plants. One day before measurements leaves were detached, glued to the base of a chamber (adaxial site), peeled off (abaxial epidermis) and left for recovery overnight in a standard solution containing 0.1 mM KCl, 1 mM $CaCl_2$ and 5 mM MES adjusted to pH 5.5-5.7 with Tris. During experiments, exposed tissue was constantly perfused with the standard solution (2 ml/min); elicitors were applied for 2 min. For impalements, microelectrodes from borosilicate glass capillaries with filament (Hilgenberg, Malsfeld, Germany) were pulled on a horizontal laser puller (P2000, Sutter Instruments Co, Novato, CA, USA). They were filled with 300 mM KCl and connected via a Ag/AgCl half-cell to a headstage (Axon Inst., Union City, CA, USA) (Scherzer *et al.*, 2015). The tip-resistance was about 20-50 MΩ, while the input resistance of the headstage was 1013 Ω. The cells were impaled using an electronic micromanipulator (MM3C, Kleindiek Nanotechnik, Germany). All recordings were amplified with an IPA-2 amplifier (Applicable Electronics, Inc., Forestdale, MA, USA) and stored on a PC (WinEDR software; Windows Electrophysiology Disk Recorder).

Calcium Detection

Polyamide meshes (4 x 1.5 cm, Th. Geyer) with a mesh size of 100 µm were placed on half-strength MS agar plates. Sterile seeds (*R-GECO1* (Keinath *et al.*, 2015)) were positioned in a thin row on the upper part of the meshes and grown in an upright position under long day conditions (16 h of light at 21°C, 8 h dark at 18°C). Seedlings were imaged 7-10 days after germination by taking the meshes and placing them in a 1-well chamber on coverglass filled with 2 mL 1/2 MS medium. Treatments were applied by replacing the 1/2 MS medium with 1/2 MS medium containing 10 µM nlp20 or flg22. Imaging of R-GECO1 fluorescence was performed with a time interval of 1 s on a Nikon SMZ18 stereo microscope equipped with an ORCA-Flash 4.0 V2 sCMOS camera (Hamamatsu, Japan), using a filter-set for red fluorescence excitation and detection (excitation: ETS 545/25x; emission: ET605/70m).

Callose Deposition

To visualize callose apposition, leaves of 5-week-old Arabidopsis plants were infiltrated with water or the indicated peptides and stained with aniline blue after 24 hours as described (Wang *et al.*, 2009). Pictures were taken with an inverted microscope (Nikon ECLIPSE 80i), then adjusted and analyzed using Photoshop CS5. Quantification of callose was performed by counting dark pixels selected by Magic tool and calculated in % relative to the total pixels of the image.

Ion Leakage Assay

Ion leakage assays were performed as described (Lenarčič *et al.*, 2017). Leaves of 5 to 6-week-old Arabidopsis plants were infiltrated with ddH_2O or 500 nM PG3. After 10 min of infiltration, leaf discs (Ø 7 mm) were punched out and transferred into a 24-well plate. Two leaf pieces per well were floated on 1 mL ddH_2O and shaken at 50 rpm. After 30 min of incubation, leaf pieces were transferred to fresh ddH_2O and conductivity was measured at the times indicated using a conductivity meter (QCond2200).

Indole glucosinolate glucobrassicin determination

Leaves of 5-week-old Arabidopsis plants were infiltrated with peptide solution or ddH_2O. For analysis of I3M (glucobrassicin) 200 mg of fresh plant leaves were harvested and homogenized in liquid nitrogen. Extraction of the analytes was carried out with 500 µl 80 % methanol containing 0.1 % formic acid, followed by a second extraction with 500 µl 20 % methanol containing 0.1 % formic acid. Both supernatants were combined and dried in a speed vac. For analysis with a Waters Acquity UPLC – SynaptG2 LC/MS system the samples were redissolved in 100 µl 20 % methanol containing 0.1 % formic acid. 5 µl were injected onto a Water Acquity HSS T3 reverse phase column. Separation was carried out with a linear 10 min 99 % water to 99% methanol (both solvents containing 0.1 % formic acid) gradient. For detection, the mass spectrometer was operated in negative ESI mode. For quantification of I3M, integrated extracted ion chromatograms were calculated into pmol with a calibration function between 1 nM and 1 mM. The obtained results were then normalized to the exact amount of fresh weight material used.

Western Blot Analyses

For MAPK activity assays, Arabidopsis leaves were infiltrated with ddH_2O or peptide solution and harvested at the indicated time points. Protein extraction and immunoblot analyses using the anti-phospho p44/42 MAP kinase antibody (Cell Signaling Technology) were performed as described (Brock *et al.*, 2010). Protoplasts were isolated using the protocol as described (Lu *et al.*, 2011). For BIK1 phosphorylation assays, 0.1 mL protoplasts at a density of 2×10^5/ml were transfected with 20 µg of plasmid DNAs carrying *BIK1-HA* as described (Lu *et al.*, 2010), then treated with flg22 or nlp20. Anti-HA antibody was used for immunoblot analyses.

Statistical analysis

Data sets were analyzed using Microsoft Office Excel or JMP® 12.2.0. Comparisons between two groups were made using Student's *t*-test. Multiple groups were compared using ANOVA followed by Student's *t*-test for all possible individual comparisons.

Accession Numbers

Sequence data from this article can be found in the Arabidopsis Genome Initiative or GenBank/EMBL databases under the following accession numbers: *AGB1*, At4g34460; *AGG1*, At3G63420; *AGG2*, At3G22942; *BAK1*, At4g33430; *BIK1*, At2g39660; *BIR2*, At3g28450; *CPK28*, At5g66210; *CYP71A13*, At2g30770; *CYP81F2*, At5g57220; *EF-1α*, At1g07920/30/40; *EFR*, At5g20480; *FLS2,* At5g46330; *MLO12*, At2g39200; *PAD3*, At3g26830; *PBL1*, At3g55450; *PP2A-A1*, At1g25490; *PP2A-C4*, At3g58500; *PR1*, At2g14610; *RLP23*, At2g32680; *RLP42*, At3g25020; *SOBIR1*, At2g31880; *XLG2*, At4g34390.

References

Albert I, Böhm H, Albert M, Feiler CE, Imkampe J, Wallmeroth N, Brancato C, Raaymakers TM, Oome S, Zhang H, Krol E, Grefen C, Gust AA, Chai J, Hedrich R, Van den Ackerveken G, Nürnberger T. 2015. An RLP23–SOBIR1–BAK1 complex mediates NLP-triggered immunity. *Nat Plants* **1**: 15140.

Böhm H, Albert I, Oome S, Raaymakers TM, Van den Ackerveken G, Nürnberger T. 2014b. A conserved peptide pattern from a widespread microbial virulence factor triggers pattern-induced immunity in Arabidopsis. *PLoS Pathog* **10**: e1004491.

Brock AK, Willmann R, Kolb D, Grefen L, Lajunen HM, Bethke G, Lee J, Nürnberger T, Gust AA. 2010. The Arabidopsis mitogen-activated protein kinase phosphatase PP2C5 affects seed germination, stomatal aperture, and abscisic acid-inducible gene expression. *Plant Physiol* **153**: 1098-1111.

Ding L, Pandey S, Assmann SM. 2008. Arabidopsis extra-large G proteins (XLGs) regulate root morphogenesis. *Plant J* **53**: 248-263.

Felix G, Duran JD, Volko S, Boller T. 1999. Plants have a sensitive perception system for the most conserved domain of bacterial flagellin. *Plant J* **18**: 265-276.

Gao M, Wang X, Wang D, Xu F, Ding X, Zhang Z, Bi D, Cheng YT, Chen S, Li X, Zhang Y. 2009. Regulation of cell death and innate immunity by two receptor-like kinases in Arabidopsis. *Cell Host Microbe* **6**: 34-44.

Halter T, Imkampe J, Mazzotta S, Wierzba M, Postel S, Bucherl C, Kiefer C, Stahl M, Chinchilla D, Wang X, Nürnberger T, Zipfel C, Clouse S, Borst JW, Boeren S, de Vries SC, Tax F, Kemmerling B. 2014. The leucine-rich repeat receptor kinase BIR2 is a negative regulator of BAK1 in plant immunity. *Curr Biol* **24**: 134-143.

Heese A, Hann DR, Gimenez-Ibanez S, Jones AM, He K, Li J, Schroeder JI, Peck SC, Rathjen JP. 2007. The receptor-like kinase SERK3/BAK1 is a central regulator of innate immunity in plants. *Proc Natl Acad Sci U S A* **104**: 12217-12222.

Kars I, Krooshof GH, Wagemakers L, Joosten R, Benen JA, van Kan JA. 2005. Necrotizing activity of five *Botrytis cinerea* endopolygalacturonases produced in *Pichia pastoris*. *Plant J* **43**: 213-225.

Keinath NF, Waadt R, Brugman R, Schroeder JI, Grossmann G, Schumacher K, Krebs M. 2015. Live cell imaging with R-GECO1 sheds light on flg22- and chitin-induced transient $[Ca^{2+}]_{cyt}$ patterns in Arabidopsis. *Mol Plant* **8**: 1188-1200.

Kunze G, Zipfel C, Robatzek S, Niehaus K, Boller T, Felix G. 2004. The N terminus of bacterial elongation factor Tu elicits innate immunity in Arabidopsis plants. *Plant Cell* **16**: 3496-3507.

Laluk K, Luo H, Chai M, Dhawan R, Lai Z, Mengiste T. 2011. Biochemical and genetic requirements for function of the immune response regulator BOTRYTIS-INDUCED KINASE1 in plant growth, ethylene signaling, and PAMP-triggered immunity in Arabidopsis. *Plant Cell* 23: 2831–2849.

Lenarčič T, Albert I, Böhm H, Hodnik V, Pirc K, Zavec AB, Podobnik M, Pahovnik D, Žagar E, Pruitt R, Greimel P, Yamaji-Hasegawa A, Kobayashi T, Zienkiewicz A, Gömann J, Mortimer JC, Fang L, Mamode-Cassim A, Deleu M, Lins L, Oecking C, Feussner I, Mongrand S, Anderluh G, Nürnberger T. 2017. Eudicot plant-specific sphingolipids determine host selectivity of microbial NLP cytolysins. *Science* **358**: 1431-1434.

Liang X, Ding P, Lian K, Wang J, Ma M, Li L, Li L, Li M, Zhang X, Chen S, Zhang Y, Zhou J-M. 2016. Arabidopsis heterotrimeric G proteins regulate immunity by directly coupling to the FLS2 receptor. *Elife* **5**: e13568.

Lin W, Li B, Lu D, Chen S, Zhu N, He P, Shan L. 2014. Tyrosine phosphorylation of protein kinase complex BAK1/BIK1 mediates Arabidopsis innate immunity. *Proc Natl Acad Sci U S A* **111**: 3632-3637.

Lu D, Lin W, Gao X, Wu S, Cheng C, Avila J, Heese A, Devarenne TP, He P, Shan L. 2011. Direct ubiquitination of pattern recognition receptor FLS2 attenuates plant innate immunity. *Science* **332**: 1439-1442.

Lu D, Wu S, Gao X, Zhang Y, Shan L, He P. 2010. A receptor-like cytoplasmic kinase, BIK1, associates with a flagellin receptor complex to initiate plant innate immunity. *Proc Natl Acad Sci U S A* **107**: 496-501.

Monaghan J, Matschi S, Shorinola O, Rovenich H, Matei A, Segonzac C, Malinovsky FG, Rathjen JP, MacLean D, Romeis T, Zipfel C. 2014. The calcium-dependent protein kinase CPK28 buffers plant immunity and regulates BIK1 turnover. *Cell Host Microbe* **16**: 605-615.

Perraki A, DeFalco T, Derbyshire P, Avila J, Séré D, Sklenar J, Qi X, Stransfeld L, Schwessinger B, Kadota Y, Macho AP, Jiang S, Couto D, Torii KU, Menke FLH and Zipfel C. 2018. Phosphocode-dependent functional dichotomy of a common co-receptor in plant signaling. *Nature*, **561**: 248-252.

Scherzer S, Bohm J, Krol E, Shabala L, Kreuzer I, Larisch C, Bemm F, Al-Rasheid KA, Shabala S, Rennenberg H, Neher E, Hedrich R. 2015. Calcium sensor kinase activates potassium uptake systems in gland cells of Venus flytraps. *Proc Natl Acad Sci U S A* **112**: 7309-7314.

Schwessinger B, Roux M, Kadota Y, Ntoukakis V, Sklenar J, Jones A, Zipfel C. 2011. Phosphorylation-dependent differential regulation of plant growth, cell death, and innate immunity by the regulatory receptor-like kinase BAK1. *PLoS Genet* **7**: e1002046.

Segonzac C, Macho AP, Sanmartin M, Ntoukakis V, Sanchez-Serrano JJ, Zipfel C. 2014. Negative control of BAK1 by protein phosphatase 2A during plant innate immunity. *EMBO J* **33**: 2069-2079.

Trusov Y, Rookes JE, Tilbrook K, Chakravorty D, Mason MG, Anderson D, Chen JG, Jones AM, Botella JR. 2007. Heterotrimeric G protein gamma subunits provide functional selectivity in Gβγ dimer signaling in Arabidopsis. *Plant Cell* **19**: 1235-1250.

Ullah H, Chen JG, Temple B, Boyes DC, Alonso JM, Davis KR, Ecker JR, Jones AM. 2003. The beta-subunit of the Arabidopsis G protein negatively regulates auxin-induced cell division and affects multiple developmental processes. *Plant Cell* **15**: 393-409.

Wang L, Tsuda K, Sato M, Cohen JD, Katagiri F, Glazebrook J. 2009. Arabidopsis CaM binding protein CBP60g contributes to MAMP-induced SA accumulation and is involved in disease resistance against *Pseudomonas syringae*. *PLoS Pathog* **5**: e1000301.

Zhang J, Li W, Xiang T, Liu Z, Laluk K, Ding X, Zou Y, Gao M, Zhang X, Chen S, Mengiste T, Zhang Y, Zhou JM. 2010. Receptor-like cytoplasmic kinases integrate signaling from multiple plant immune receptors and are targeted by a *Pseudomonas syringae* effector. *Cell Host Microbe* **7**: 290-301.
